# Adolescent Tuning of Association Cortex in Human Structural Brain Networks

**DOI:** 10.1093/cercor/bhx249

**Published:** 2017-10-27

**Authors:** František Váša, Jakob Seidlitz, Rafael Romero-Garcia, Kirstie J Whitaker, Gideon Rosenthal, Petra E Vértes, Maxwell Shinn, Aaron Alexander-Bloch, Peter Fonagy, Raymond J Dolan, Peter B Jones, Ian M Goodyer, the NSPN consortium, Olaf Sporns, Edward T Bullmore

**Affiliations:** 1 Brain Mapping Unit, Department of Psychiatry, University of Cambridge, Cambridge CB2 0SZ, UK; 2 Developmental Neurogenomics Unit, National Institute of Mental Health, Bethesda, MD 20892, USA; 3 The Alan Turing Institute for Data Science, British Library, London NW1 2DB, UK; 4 Department of Brain and Cognitive Sciences, Ben-Gurion University of the Negev, PO Box 653, Beer-Sheva 8410501, Israel; 5 Department of Psychiatry, Yale University School of Medicine, New Haven, CT 06511, USA; 6 Research Department of Clinical, Educational and Health Psychology, University College London, London WC1E 6BT, UK; 7 Wellcome Trust Centre for Neuroimaging, UCL Institute of Neurology, University College London, London WC1N 3BG, UK; 8 Max Planck University College London Centre for Computational Psychiatry and Ageing Research, University College London, London WC1B 5EH, UK; 9 Cambridgeshire & Peterborough NHS Foundation Trust, Huntingdon PE29 3RJ, UK; 10 Department of Psychological and Brain Sciences, Indiana University, Bloomington, IN 47405, USA; 11 Immunology & Inflammation Therapeutic Area Unit, GlaxoSmithKline R&D, Stevenage SG1 2NY, UK

**Keywords:** adolescence, connectome, development, graph theory, MRI

## Abstract

Motivated by prior data on local cortical shrinkage and intracortical myelination, we predicted age-related changes in topological organization of cortical structural networks during adolescence. We estimated structural correlation from magnetic resonance imaging measures of cortical thickness at 308 regions in a sample of *N* = 297 healthy participants, aged 14–24 years. We used a novel sliding-window analysis to measure age-related changes in network attributes globally, locally and in the context of several community partitions of the network. We found that the strength of structural correlation generally decreased as a function of age. Association cortical regions demonstrated a sharp decrease in nodal degree (hubness) from 14 years, reaching a minimum at approximately 19 years, and then levelling off or even slightly increasing until 24 years. Greater and more prolonged age-related changes in degree of cortical regions within the brain network were associated with faster rates of adolescent cortical myelination and shrinkage. The brain regions that demonstrated the greatest age-related changes were concentrated within prefrontal modules. We conclude that human adolescence is associated with biologically plausible changes in structural imaging markers of brain network organization, consistent with the concept of tuning or consolidating anatomical connectivity between frontal cortex and the rest of the connectome.

Human adolescence is known to be a major phase of cortical development. In particular, cerebral cortex becomes thinner (Wierenga et al. 2014) and more densely myelinated (Miller et al. 2012) in the transition from puberty to young adulthood. Adolescent decreases in cortical thickness (CT) (thinning) are variable between different areas of cortex (Raznahan et al. 2011), for example, thinning is greater in association cortical areas than primary sensory areas (Whitaker, Vértes et al. 2016).

Motivated by these and other results, we predicted that human adolescence should be associated with changes in the architecture of structural brain networks. There are currently only two experimental techniques, both based on magnetic resonance imaging (MRI), that are capable of providing data to test this prediction: diffusion tensor imaging followed by tractography; or structural MRI followed by structural covariance or correlation analysis. Here we focused on the latter, measuring the thickness of a set of predefined cortical regions in each individual MRI dataset and then estimating the correlation of thickness between each possible pair of regions across participants. Similar methods have been widely used and validated (Lerch et al. 2006) in a range of prior studies (Alexander-Bloch, Giedd et al. 2013; Evans 2013).

In particular, structural correlation (covariance) measures have been used as a basis for graph theoretical modeling of the human connectome (Bullmore and Sporns 2009; Fornito et al. 2016). Considerable evidence has accumulated in support of the general view that human brain structural correlation networks have a complex topological organization, characterized by nonrandom features such as the existence of highly connected (high degree) hub nodes and a modular community structure (Alexander-Bloch, Giedd et al. 2013; Evans 2013). Topological metrics on structural correlation networks have demonstrated changes associated with disease, development, and ageing (Alexander-Bloch, Giedd et al. 2013; Evans 2013). However, only two studies have investigated adolescent changes in structural correlation networks. Zielinski et al. (2010) demonstrated that the anatomical extent of structural correlation networks, assessed using seed-based correlation of voxel-wise grey matter intensity, changes in adolescence in a spatially patterned manner. Specifically, primary visual and sensorimotor networks, as well as the default mode network, expanded in early childhood before being “pruned” in adolescence, while higher-order cognitive networks showed a gradual monotonic gain in spatial extent. Subsequently, Khundrakpam et al. (2013) applied graph-theoretical analyses to a subset of the same data, reporting childhood increases in topological integration (global efficiency) and decreases in topological segregation (local efficiency and modularity), as well as increases in regional integration in paralimbic and association regions. While these studies constitute interesting initial investigations, their ability to precisely describe developmental changes is limited by their segregation of participants into four discrete age-defined strata, resulting in relatively coarse-grained resolution of brain maturational trajectories.

Here, we aimed to obtain a more precise description of adolescent maturational trajectories of structural network architecture, which were hypothesized to vary as a smooth and potentially nonlinear function of age. We used a sliding-window analysis to estimate structural correlations and structural network properties for each of an overlapping series of nine age-defined windows or strata of the sample (*N* ≈ 60 participants per window). We identified the cortical regions (nodes) and connections (edges) which showed the most significant age-related changes in structural correlation. We tested the related hypotheses that parameters of adolescent change in structural correlation would be greater and occur later in regions of association cortex, which show faster rates of local cortical shrinkage and myelination. In addition, we explored whether greater and later changes in structural correlation during adolescence would be concentrated within or between specific communities of regions. Specifically we mapped adolescent changes in structural correlation to three brain community structures: the topological modular partition of the age-invariant structural correlation network; an atlas of cytoarchitectonic classes (von Economo and Koskinas 1925); and functional intrinsic connectivity or resting state networks (Yeo, Krienen et al. 2011).

## Materials and Methods

### Participants

A demographically balanced cohort of 297 healthy participants (149 females) aged 14–24 years was included in this study, with approximately 60 participants in each of 5 age-defined strata: 14–15 years inclusive, 16–17, 18–19, 20–21, and 22–24 years. Participants were excluded if they were currently being treated for a psychiatric disorder or for drug or alcohol dependence; had a current or past history of neurological disorders or trauma; or had a learning disability. Participants provided informed written consent for each aspect of the study, and parental consent was obtained for those aged 14–15 years. The study was ethically approved by the National Research Ethics Service and was conducted in accordance with NHS research governance standards.

### MRI Acquisition and Processing

Structural scans were acquired at three sites using multiparametric mapping (MPM) implemented on three identical 3 T MRI scanners (Siemens Magnetom TIM Trio). Intersite reliability of the sequence was evaluated within a pilot study of 5 healthy participants each scanned at each site (Weiskopf et al. 2013). The MPM sequence includes maps of *R*_1_ (1/*T*_1_) and magnetization transfer (MT), indicative of myelination. For details of MRI acquisition parameters, see Supplementary Information.

Processing of individual scans using FreeSurfer v5.3.0 included skull-stripping, segmentation of cortical grey and white matter and reconstruction of the cortical surface and grey-white matter boundary (Fischl et al. 1999). All scans were stringently quality controlled by re-running the reconstruction algorithm after the addition of control points and white matter edits (details in Supplementary Information). The cerebral cortex of each participant was parcellated into 308 regions of interest, based on a sub-division of the Desikan-Killiany anatomical atlas (Desikan et al. 2006) into parcels of approximately equal surface area (~5 cm^2^) (Romero-Garcia et al. 2012).

Regional changes in cortical thickness (CT) and MT (myelination) were characterized using the rate of change over adolescence, evaluated as the slope of a linear model fitted to the cross-sectional values. Following Whitaker, Vértes et al. (2016), myelination analyses were conducted at 10 fractional depths between the pial surface and the grey/white matter boundary, as well as 2 absolute depths into white matter. Main analyses focused on MT estimates at 70% fractional cortical depth from the pial surface. For details and results across cortical depths, see the Supplementary Information.

While both CT and myelination maps were averaged within parcels, for comparison between maturation of structural correlation networks and morphology, only the CT values were used to construct structural correlation networks.

### Age-Invariant Structural Network

**Figure 1.**
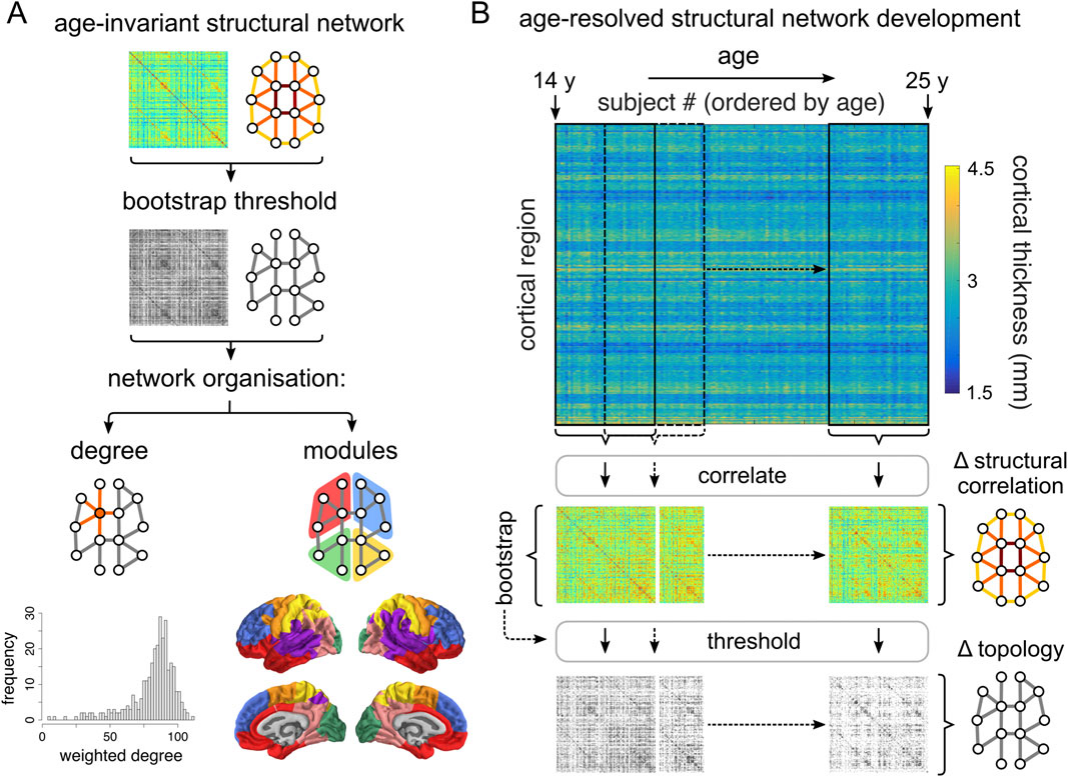
Construction of age-invariant and age-resolved structural correlation networks. (*A*) An age-invariant structural correlation network was constructed by cross-correlating regional cortical thickness across all participants. This network was probabilistically thresholded using a bootstrap-based method. Network organization was evaluated using several measures, including the degree (both binary and weighted; respectively the number and sum of weights of retained edges connected to a node) and modular architecture. For details regarding module generation, see Supplementary Information (Fig. S1). (*B*) Age-resolved structural correlation networks were constructed using a sliding-window method. Participants were ordered by age, and structural networks were constructed by estimating correlations between regional cortical thickness values across participants within overlapping windows iteratively slid across the age range. Correlations were probabilistically thresholded using bootstrap, before developmental trajectories were fitted to summary window-derived measures as a function of the median age of participants within each window.

An age-invariant structural correlation network was constructed using Pearson correlations in CT between pairs of regions across all 297 participants, to serve as a reference for developmental changes within the age-resolved structural networks (described below; Fig. 1*A*). We used raw CT values, uncorrected for age, gender, or intracranial volume. However, correcting for these covariates had no effect on the results. For background reading on graph theoretical methods and connectomics see Bullmore and Sporns (2009) and Fornito et al. (2016).

The age-invariant structural network was thresholded using a bootstrap approach, whereby 1000 sets of participants were resampled with replacement and used to construct surrogate structural networks. For each pair of regions, we examined whether there is evidence of a nonzero correlation across bootstraps: edges that were consistently positive or negative across bootstraps (at a two-tailed, false discovery rate [FDR]-adjusted level of *α* = 0.01) were retained; the remaining edges were set to zero. Nodal topological organization of the thresholded network was assessed using degree, defined as the number of retained correlations for each node, as well as the weighted degree, or summed weight of retained edges for each node.

Further, the age-invariant network was partitioned into communities of nodes showing higher structural correlations within than between communities (Sporns and Betzel 2016). The community structure of the age-invariant network was decomposed using the Louvain multiresolution algorithm (Blondel et al. 2008) over the resolution parameter range 0.01 ≤ *γ* ≤ 4.00. As *γ* increases, the community structure is decomposed to a progressively larger number of modules. We used the concept of minimizing versatility to identify those resolution parameter values which reduce the uncertainty with which any node was affiliated consistently to the same module (Shinn et al. 2017). The final community partition was defined as a consensus across 1000 runs of the Louvain modularity algorithm (Lancichinetti and Fortunato 2012) at the selected value of the resolution parameter *γ*. For details regarding module generation, see Figure S1.

### Development of Age-Resolved Structural Networks

#### Sliding Window Network Construction

Development of structural networks between 14 and 24 years was evaluated using a sliding window method. Regional CT values were cross-correlated within windows containing equal numbers of participants, and incrementally slid across the age-range by regular increments (Fig. 1*B*). The two parameters of the method, the “window width” and the “step size” (in units of number of participants) determine the number of windows, each of which generates a structural correlation network. Exploration of the sliding window parameter values suggests that results are qualitatively consistent across a range of parameter combinations. For the (in)dependence of results on sliding window parameters, and a discussion of the considerations involved in parameter selection, see the Supplementary Information.

Results presented below correspond to 9 half-overlapping windows of 60 participants each, obtained by interpolating the 5 age strata of the NSPN study, within which participants were recruited. Gender was relatively balanced within the interpolated bins, with the most imbalanced ratio being 34:26 = 57%:43% (M:F). We investigated the effects of gender separately (see below).

Global maturation of structural networks was characterized using the mean of the correlation distribution. At the regional level, an analogous measure was used—nodal strength, the mean of the pattern of regional correlations (rows, or equally, columns of the correlation matrices).

#### Bootstrap Thresholding of Age-Resolved Structural Networks

**Figure 2.**
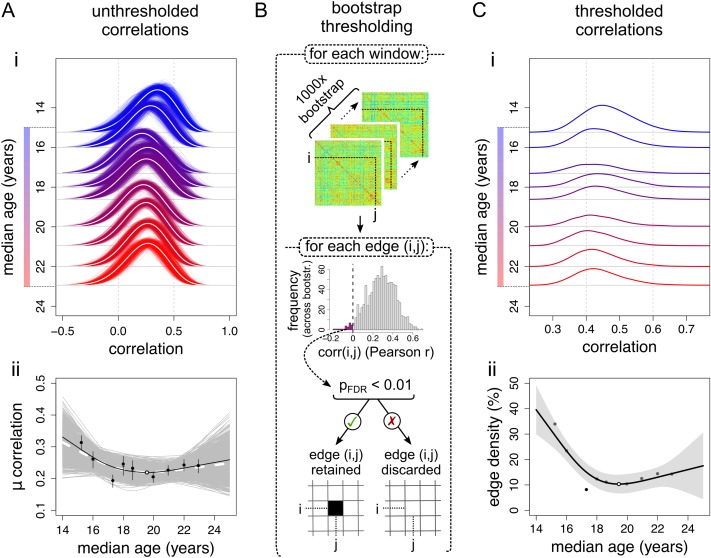
Global trajectories of age-resolved structural correlations and network connection density. (*A*) Global trajectories of unthresholded structural correlations. (i) Development of the distribution of unthresholded correlations across age windows. Thin lines represent bootstrapped estimates, white lines represent the bootstrap mean. (ii) Changes in the average correlation. Black markers represent empirical data (error bars indicate the interquartile range across bootstraps), with corresponding regression line; the white marker indicates the trajectory minimum. Grey lines represent bootstrapped trajectories; the white dashed line represents the bootstrap mean. (*B*) Each windowed matrix was thresholded using bootstrap. Within each window, 1000 sets of participants were resampled (with replacement) and used to construct correlation matrices. For each edge (correlation) within each window, the presence of a significant nonzero correlation (across bootstraps) was tested at the FDR-adjusted level of *α*_FDR_ = 0.01. Consistent correlations were retained, while inconsistent correlations were assigned a value of 0. (*C*) Global trajectories within thresholded structural correlation networks. (i) Development of the distribution of correlations retained after probabilistic thresholding across age windows. (ii) The number of edges retained after probabilistic thresholding, or edge density. The shaded area represents the 95% confidence interval of the spline fit.

Estimating structural correlation networks from a small number of participants is an inherently noisy process; therefore, our principal analyses focused on networks probabilistically thresholded using bootstrap (Fig. 2*B*). The bootstrap thresholding procedure was identical to the one described above for age-invariant networks, but in this case was applied within windows. From the set of participants included in each window, an equal number of participants was sampled with replacement and the correlation structure was re-estimated 1000 times. For each pair of regions, we examined whether there is evidence of a nonzero correlation across bootstraps: edges that were consistently positive across bootstraps (at a two-tailed, FDR-adjusted level of *α* = 0.01) were retained (there were no consistently negative edges); the remaining edges were set to zero.

The global topological organization of the thresholded graphs was assessed using the edge density, defined as the percentage of retained edges (relative to their possible total), as well as the distance spanned by retained edges, calculated as the average Euclidean distance between centroids of corresponding nodes. Nodal topological organization was assessed using (analogous) measures of degree, defined as the number of edges connected to a node, and average Euclidean distance spanned by a node’s retained edges. We have focused on simple graph-theoretical measures, such as edge density and node degree, for two reasons: (1) our bootstrap-thresholded networks display variable edge density, which many “higher-order” graph-theoretical measures show a strong dependence on (van Wijk et al. 2010), and (2) even in correlation-based networks thresholded to fixed edge density, graph theoretical properties display a dependence on more elementary statistics such as properties of the correlation distribution (van den Heuvel et al. 2017).

### Fitting and Characterization of Developmental Trajectories

Developmental trajectories were fitted to both global and local measures as a function of the median age of participants in each window. In addition to linear models, we fitted locally adaptive smoothing splines. The nonparametric smoothing spline was chosen to model nonlinear trajectories over parametric alternatives as it was shown to be superior to quadratic fits in studies of brain development (Fjell et al. 2010). Still, the spline fits were constrained to be (approximately) at least as smooth as a quadratic fit (i.e., effective degrees of freedom, *df* ≤ 3.5), based on the hypothesis that adolescent developmental trajectories over a 10-year age range should not display greater complexity. The specific smoothing spline used was a weighted sum of 6 cubic b-splines with knots placed at quantiles of the data and smoothing optimized using restricted maximum likelihood (REML) (Reiss et al. 2014). The relative quality of linear and spline fits, given their parsimony, was assessed using Akaike’s information criterion (AIC). Classification using the Bayesian information criterion (BIC) yielded consistent results.

Regional changes were summarized using measures of maximum change in degree Δ*k*_max_, quantified as the difference between maximum and minimum degree, and the age at minimum degree age(*k*_min_). Further, we classified regional changes in degree as linear or nonlinear (using the AIC), and as increasing or decreasing (using the direction of maximum change). As an alternative measure of the magnitude of regional changes in structural correlation, we extracted linear rates of change of degree; the results were qualitatively consistent with the measure of maximum change, which is more suitable for nonlinear trajectories (Supplementary Information).

### Relationship of Structural Network Development to Age-Invariant Network Architecture

Given our previous finding, that highly correlated “hub nodes” of the age-invariant structural network (derived from all participants) are regions which thin and myelinate most over adolescence (Whitaker, Vértes et al. 2016), we were interested in studying the relationship of structural network development to age-invariant structural network architecture.

We evaluated Spearman’s rank correlations between node degree in the age-invariant structural network, and parameters of change in node degree within the age-resolved structural network—including the amplitude of maximum change in degree Δ*k*_max_ as well as the age at minimum degree age(*k*_min_).

Finally, we studied changes in structural network organization relative to three sets of node communities, including the partition of the age-invariant network into modules, the von Economo atlas of cytoarchitectonic classes (von Economo and Koskinas 1925), and a set of functional intrinsic connectivity networks (Yeo, Krienen et al. 2011). For each community template and each age-window, we calculated the density of edges, *D*, within each community as well as between each pair of communities (within the same template), as the ratio of existing edges relative to the maximum number of possible edges in this within or between-community edge set. We then characterized changes in edge density within and between communities using measures analogous to the nodal trajectories—maximum change in edge density Δ*D*_max_ and age at minimum density age(*D*_min_). For details regarding the matching of the community templates to our 308-region parcellation, see the Supplementary Information.

### Spatial Permutation Test

In several analyses in the current study, measures were related to each other across regions. While numerous studies have reported significance based on the assumption that the number of samples is equal to the number of regions, this is technically inaccurate, as the number of regions is both arbitrary (due to the resolution of the chosen parcellation) and non-independent (due to spatial autocorrelation amongst neighboring parcels). To address this issue, spatial permutation tests have been implemented in past studies (Alexander-Bloch, Giedd et al. 2013; Alexander-Bloch, Raznahan et al. 2013; Vandekar et al. 2015), which consist in comparing the empirical correlation amongst two spatial maps to a set of null correlations, generated by randomly rotating the spherical projection of one of the two spatial maps (as generated in FreeSurfer or Caret) before projecting it back on the brain surface. Importantly, the rotated projection preserves spatial contiguity of the empirical maps, as well as hemispheric symmetry. Such tests were previously implemented at the vertex level (Alexander-Bloch, Giedd et al. 2013; Alexander-Bloch, Raznahan et al. 2013; Vandekar et al. 2015); here we implemented an analogous permutation test at the regional level. Thus, each analysis correlating values from two cortical maps is reported with both the *P*-value corresponding to the Spearman correlation (*P*_Spearman_), as well as a *P*-value derived from the spherical permutation (*P*_perm_), obtained by comparing the empirical Spearman’s *ρ *to a null distribution of 10 000 Spearman correlations, between one empirical map and the randomly rotated projections of the other map. For full details on the spherical permutation test, see Supplementary Information.

### Sensitivity Analyses

To ascertain the robustness of obtained results to sliding window parameters and other methodological decisions and to rule out effects of potential artefactual causes, we conducted several ancillary studies.

We first investigated effects of sliding window parameters by systematically varying the window width and step size over ranges of {40,60,80} and {5,10,20} participants, respectively.

Further, we examined potential effects of gender by repeating sliding window analyses separately for each gender (149 female and 148 male participants). This resulted in 9 windows of ~30 participants each. Following estimation of global and nodal sliding window statistics separately for each gender within both unthresholded and bootstrap-thresholded networks (as described for all participants above), we fitted linear and spline models to the combined data, separately modeling effects of age, gender, and the age-by-gender interaction.

Finally, we studied the effect of several potential artefacts, including the presence of regions with low reliability of structural correlations as well as irregularities in the age distribution of participants.

For full results and discussion of these additional studies, see Supplementary Information.

## Results

### Age-Invariant Structural Network

We first considered the structural correlation network constructed by thresholding the pairwise inter-regional correlations estimated from CT measurements on all (297) participants, age range 14–24 years (inclusive). Since this analysis combines data from all ages in the sample, we can refer to the result as an age-invariant structural correlation network (Fig. 1*A*).

The distribution of structural correlations had a positive mean value and was approximately symmetrical. The structural correlation matrix was thresholded probabilistically, using a bootstrap-based resampling procedure (Materials and Methods), to control the edge-wise false positive rate. Since this thresholding operation entailed approximately 47 000 hypothesis tests, we used the FDR algorithm to adjust for multiple comparisons. The resulting graph was densely connected (connection density ≈ 90%) and exhibited a modular community structure (Fig. 1*A*). The community partition consisted of 7 modules, including three primary cortex modules: somatosensory (anterior parietal cortex), motor (posterior frontal cortex), and visual (occipital cortex), as well as an inferior-frontal/temporal module, a superior frontal module, a superior temporal/insular module and a parieto-occipital module. For details on this community structure and other modular partitions comprising different numbers of modules see Figure S1 and Table S1.

### Age-Resolved Structural Networks

To resolve age-related changes in structural networks, we used a “sliding window” analysis to estimate the structural correlation matrix separately for each of a series of subsets of the sample defined by overlapping age ranges or windows (Fig. 1*B*). The results of this analysis are naturally somewhat dependent on the sliding window parameters: the age-range spanned by each window and the incremental step between windows. Below we focus on results obtained with 9 windows of ~60 participants each, ranging from [14.1–16.0 years] to [22.0–25.0 years] with an incremental step of 30 participants (~1 year). We also explored a range of alternative sliding window parameters and demonstrated that our key results were robust to this methodological variation (Supplementary Information).

Globally, over the whole brain, there was a nonlinear trend of reducing structural correlation from the youngest age window to the oldest age window (Fig. 2*A*). Relatively strong positive correlations at age 14 (>0.31) decreased sharply over the next few windows, with minimum mean correlation (~0.22) occurring at 19.59 years (95% confidence interval (CI) [19.37, 19.76] years) and then slightly increasing again towards age 24 (AIC_spl_ < AIC_lin_, *r*^2^_adj_ = 0.52, *P* = 0.098; Fig. 2*A*ii). Both the mean inter-regional covariance, and the mean product of regional standard deviations (respectively the numerator and denominator of the Pearson correlation coefficient), showed similar nonlinear processes of decline in younger windows followed by levelling off in older windows (Fig. S4).

A potential drawback of the sliding window analysis is that it inevitably involves estimating inter-regional correlations on a subset of the sample (*N* ≈ 60 per window), with commensurately reduced precision of estimation and therefore noisier graphs. We used a probabilistic threshold to control the edge-wise FDR at 1%, thus ensuring that the age-resolved graphs only included edges that were unlikely to represent false positive noise (Fig. 2*B*).

Focusing on the most statistically robust subset of edges (which passed the FDR threshold for significance), we found similar but clearer evidence for age-related global changes in structural network organization. The structural correlation distributions of the bootstrap-thresholded network became sparser over the course of adolescence (Fig. 2*C*i). The edge density demonstrated a nonlinear decrease (AIC_spl_ < AIC_lin_) from 33.9% to a minimum of 8.2% at 19.45 years (95% CI [19.32, 19.59] years; *r*^2^_adj_ = 0.81, *P* = 0.0069), which was similar in shape to the global trajectory of unthresholded correlation (Fig. 2*C*ii).

The global connection distance of the thresholded networks (the mean Euclidean distance subtended by bootstrap-thresholded edges) also demonstrated a nonlinear trajectory (AIC_lin_ < AIC_spl_, *r*^2^_adj_ = 0.67, *P* = 0.049) characterized by a phase of relatively rapid decrease from 14 years to reach a minimum at 18.72 years (95% CI [18.68, 18.77] years), followed by a phase of more stable connection distance (Fig. S7A).

### Regional Development of Age-Resolved Structural Networks

**Figure 3.**
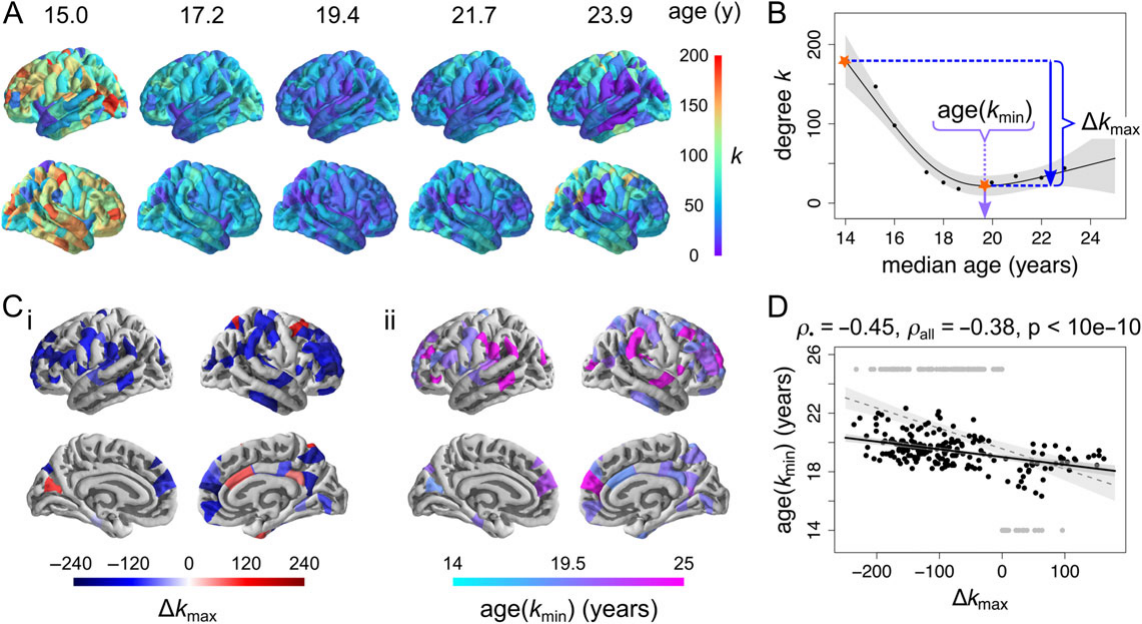
Regional development of structural correlation networks. (*A*) Cortical maps of node degree at 5 regularly sampled intervals of the developmental trajectories, showing a regionally heterogeneous decrease from young age. (*B*) Definition of local measures of maturation, illustrated on a nonlinearly decreasing trajectory (from the right dorsolateral prefrontal cortex). The maximum change in degree Δ*k*_max_ corresponds to the (absolute) difference (decrease or increase) in degree between the maximum and the minimum of the trajectory. The age at minimum degree age(*k*_min_) corresponds to the timing of the minimum of the trajectory. (*C*) Cortical maps of regional maturation measures for trajectories showing evidence of nonzero change (at *P*_FDR_ < 0.05), predominantly located in association cortex: (i) maximum change in degree, and (ii) age at minimum degree. (*D*) Regions that show greater decreases in degree tend to reach minima of their trajectories later, whether considering all regions (grey) or excluding regions where the trajectory minimum occurs at extrema of the age range (black).

Regional maturation of structural correlation networks was assessed by estimating the trajectories of changes in node degree, which is the number of correlations retained at each node (following bootstrap thresholding). Although there was regional heterogeneity in the trajectories of node degree (Fig. 3*A*), all regions that demonstrated significant evidence of nonzero change (linear or spline fit *P*_FDR_ < 0.05; 82 regions) followed a nonlinear trajectory (AIC_spl_ < AIC_lin_), which for most regions (75/82) could be summarized by a younger phase (from 14 to 19 years approximately) of more-or-less rapid decrease in structural correlation followed by a levelling off or slight increase of structural correlation in an older phase (from 19 to 24 years approximately). This process could be summarized by two parameters: Δ*k*_max_, the difference between maximum and minimum degree; and age(*k*_min_), the age at which node degree reached its minimum value (Fig. 3*B*).

Decreases in node degree were greatest in association cortical areas, such as bilateral dorsolateral prefrontal cortex, medial frontal cortex and supramarginal gyrus, as well as precentral and postcentral gyri and several temporal cortical regions. Increases in node degree were less spatially clustered, occurring in isolated nodes within the right cingulate, superior frontal and parietal cortices as well as left cuneus (Fig. 3*C*i). Association cortical areas also showed more prolonged decreases in structural correlation, reaching the minimum value of node degree later (Fig. 3*C*ii). Predictably, it follows that the extent of degree shrinkage Δ*k*_max_ was negatively correlated with the age at which degree reached its minimum value age(*k*_min_), whether considering all regions (Spearman’s *ρ* = −0.38, *P*_Spearman_ < 10^−10^, *P*_perm_ < 10^−5^) or excluding regions whose minimum occurred at one of the limits of the age range (Spearman’s *ρ* = −0.45, *P*_Spearman_ < 10^−10^, *P*_perm_ = < 10^−5^; Fig. 3*D*).

Age-related nonlinear changes in nodal connection distance (the mean Euclidean distance of all edges connecting a node within the bootstrap-thresholded network) were summarized using analogous parameters to node degree: Δ*d*_max_, the difference between maximum and minimum distance; and age(*d*_min_), the age at which nodal connection distance reached its minimum value. Nodes that demonstrated significantly reduced connection distance (*P*_FDR_ < 0.05) were located in left dorsolateral prefrontal cortex, left supramarginal gyrus and right superior parietal cortex (Fig. S7C). Decreases in node connection distance were negatively correlated with age at minimum connection distance, whether considering all nodes (Spearman’s *ρ* = −0.38, *P*_Spearman_ < 10^−10^, *P*_perm_ < 10^−5^) or excluding nodes whose minimum occurs at one of the limits of the age range (Spearman’s *ρ* = −0.25, *P*_Spearman_ = 0.0027, *P*_perm_ = 0.0036) (Fig. S7D). Finally, decreases in node connection distance were positively correlated with decreases in node degree (Spearman’s *ρ* = 0.32, *P*_Spearman_ = 1.9·10^−8^, *P*_perm_ = <10^−5^) (Fig. 7E). In other words, nodes that had the greatest reduction in hubness during adolescence also tended to have the greatest reduction in connection distance.

**Figure 4.**
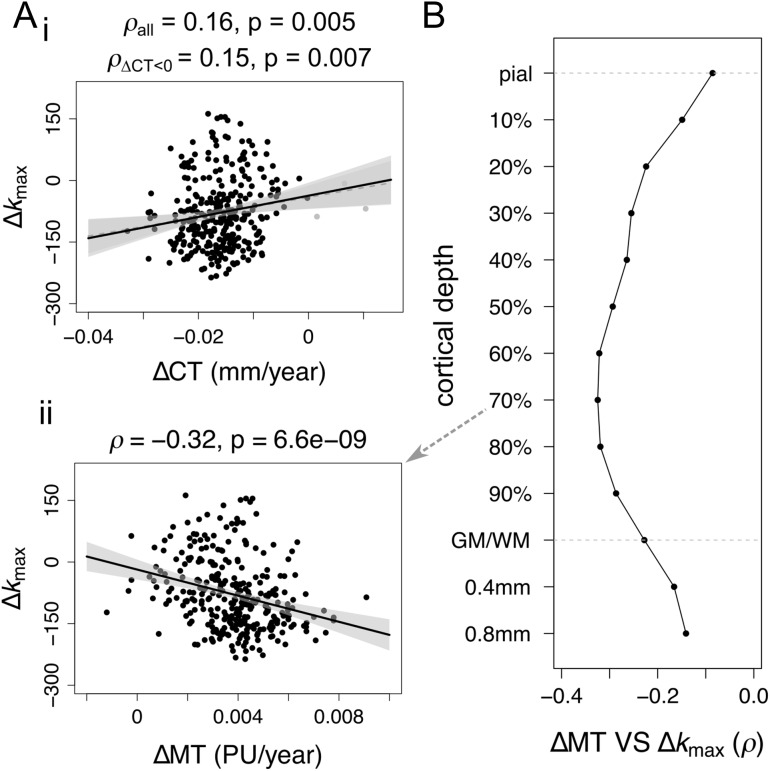
Relationship between maturation of cortical morphology and structural correlation networks. (*A*) Relationship between regional trajectories of cortical morphology and node degree. Maximum changes in nodal degree are only very weakly related to regional rates of (i) thinning and (ii) myelination (PU = percentage units). The direction of the relationships is such that cortical regions that myelinate more during adolescence are more likely to decrease in node degree and connection distance in the same period. (*B*) Spearman correlation of rate of change myelination to maximal change in degree as a function of cortical depth, including 10 fractional depths from the pial surface to the grey/white matter boundary (GM/WM), as well as 2 absolute depths into the white matter.

To contextualize changes in structural network architecture with respect to maturation of cortical morphology, we related regional measures of cortical network development to rates of change of CT and MT (a measure of myelination), evaluated as the slope of a linear model fitted to the cross-sectional values. The maximum change in node degree was (weakly) positively correlated to the rate of thinning (ΔCT; Spearman’s *ρ* = 0.16, *P*_Spearman_ = 0.0050, *P*_perm_ = 0.023; unaffected by excluding 3 outlier regions which showed ΔCT > 0, Spearman’s *ρ* = 0.15, *P*_Spearman_ = 0.0070, *P*_perm_ = 0.028; Fig. 4*A*i), and more strongly negatively correlated to the rate of intracortical myelination (ΔMT; Spearman’s *ρ* = −0.32, *P*_Spearman_ = 6.6·10^−9^, *P*_perm_ = 7·10^−4^; Fig. 4*A*ii). Following Whitaker, Vértes et al. (2016), myelination analyses were conducted at 10 fractional depths between the pial surface and the grey/white matter boundary, as well as 2 absolute depths into white matter. The strength of association between local adolescent myelination (indexed by ΔMT) and adolescent decrease of node degree (indexed by Δ*k*_max_) was greatest when ΔMT was measured at about 70% of cortical depth from the pial surface to the grey/white matter boundary (Fig. 4B).

### Age-Resolved Network Changes in Relation to the Age-Invariant Network and its Communities

Given that most densely connected nodes (hubs) of the age-invariant structural correlation network are predominantly located in association cortex (Whitaker, Vértes et al. 2016), which is also the location of greatest age-resolved decreases in structural correlation, it is not surprising that there is an inverse relationship between age-invariant (weighted) node degree and maximum change in degree (*ρ* = −0.43, *P*_Spearman_ < 10^−10^, *P*_perm_ = <10^−5^; Fig. S9Bi). Node degree of the age-invariant network and age at minimum degree were not strongly related (Fig. S9Bii).

**Figure 5.**
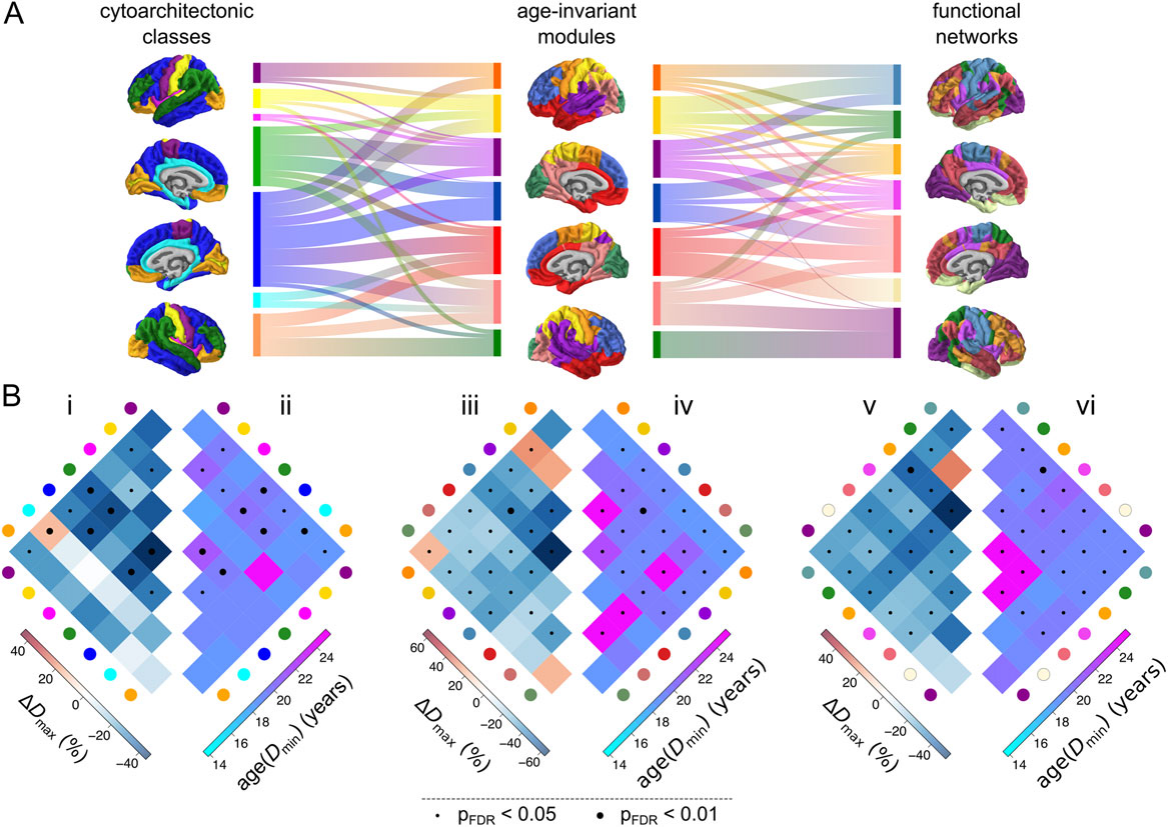
Adolescent development of structural networks in relation to human brain communities. The modular partition used consisted of 7 modules, including a parietal “somatosensory” module (yellow), a frontal “motor” module (orange), an occipital “visual” module (green), an inferior-frontal/temporal module (red), a superior frontal module (blue), a superior temporal/insular module (purple) and a parieto-occipital module (pink). (*A*) Comparison of the modular architecture of the age-invariant structural correlation network (middle) to two prior community structures—the von Economo atlas of cytoarchitectonic classes (von Economo and Koskinas 1925; left) and 7 functional intrinsic connectivity networks derived using an independent fMRI data (Yeo and Krienen 2011; right). The alluvial diagrams between surface plots of community architecture indicate the amount of overlap between individual communities across templates. (*B*) Development of structural correlations within and between corresponding pairs of communities—cytoarchitectonic classes (i, ii), age-invariant modules (iii, iv) and functional intrinsic connectivity networks (v,vi). Left: maximum change in edge density Δ*D*_max_ within and between all pairs of communities. Right: age at minimum edge density age(*D*_min_) within and between all pairs of communities. Dot markers indicate statistical significance of developmental change; small: *P*_FDR_ < 0.05, large: *P*_FDR_ < 0.01.

We further studied adolescent changes in nodal topology in relation to the community structures of the human brain. Many community structures have been proposed to partition the cortex into a set of modules or sub-networks, each comprising a number of functionally and/or anatomically related cortical areas. Here, we considered three complementary community structures: (1) the modular decomposition of the age-invariant structural correlation network (7 modules); (2) the classic von Economo cytoarchitectonic partition of the cortex into classes based on cortical lamination (we used a partition into 7 classes by Vértes et al. (2016), extended from the original partition into 5 classes by von Economo and Koskinas (1925)); and (3) the prior identification of 7 resting state networks derived from independent components analysis of an independent resting state fMRI dataset (Yeo, Krienen et al. 2011). The three classification systems had similar but not identical community structures; normalized mutual information (NMI, a measure of correspondence between two community structures) ranged from NMI = 0.39 for the relationship between the structural network modules and the resting state fMRI components to NMI = 0.29 for the relationships between both neuroimaging-based community structures and the von Economo classification (Fig. 5*A*).

In the context of (1) the age-invariant structural network community structure, the greatest decreases in connection density Δ*D*_max_ were concentrated within the superior frontal module (blue) and within the superior temporal/insular module (purple); or between the superior frontal module and other modules (Fig. 5*B*iii). The age at minimum density age(*D*_min_) tends to occur later within the same modules, as well as the occipito-parietal module (pink; Fig. 5*B*iv). In the context of (2) cytoarchitectonic atlas of von Economo and Koskinas (1925), greatest decreases in edge density were concentrated within and between association cortical areas with lamination types 2 and 3 (described as granular isocortex; blue and green respectively) and particularly within class 3 (green; Fig. 5*B*i). Association cortical trajectories tended also to reach the age of minimum edge density latest (Fig. 5*B*ii). In the context of (3) fMRI resting state networks outlined by Yeo, Krienen et al. (2011), the greatest decreases in edge density were concentrated within the frontoparietal control network (orange) as well as between this network and the other networks (Fig. 5*B*v). Minima of the trajectory were reached latest within the default mode network (salmon red) and the ventral attention network (pink), as well as between these two functional networks (Fig. 5*B*vi). In summary, across the three community partitions, the greatest (and latest) decreases in connection density occurred within association cortical communities, and (to a lesser extent) between those association cortical communities and the remainder of the network.

### Sensitivity Analyses

While we had no hypotheses about the shape of the maturational trajectories or the direction of the changes, the finding of a nonlinear decrease in structural correlation (and derived measures of edge density and degree), globally and locally, was somewhat surprising. This is one of the reasons why we conducted numerous sensitivity analyses, to ensure that our findings are not caused or inflated by methodological choices or artefacts.

Our principal findings on bootstrap-thresholded networks were corroborated by similar results from analysis of unthresholded structural correlation matrices (Fig. S6).

We evaluated robustness of our findings to parameters of the sliding window method, varying the window width and step size over ranges of {40,60,80} and {5,10,20} participants respectively. Results were qualitatively consistent with the above, showing a nonlinear decrease in structural correlation both globally and locally (most prominently in association cortex), as well as (weak) relationships of maximum local change in correlation to regional rates of thinning and myelination (Table S2 and Fig. S11).

Analysis of gender differences failed to identify effects of gender or age-by-gender interactions in the trajectories of structural correlation development (Supplementary Information).

We investigated the effect of several potential artefacts, including the presence of regions with low reliability of structural correlations (Fig. S12) as well as inhomogeneities in the age distribution of participants (Fig. S13). We found no substantial evidence that the effect of such artefacts could inflate or account for our main finding of a nonlinear age-related decrease in structural correlation.

Finally, we investigated whether subtle nonlinearities in trajectories of cortical thinning and myelination could be driving nonlinearities in trajectories of structural correlation (Figs S14–16). Although neither nonlinear CT or MT effects were especially strong, subtle nonlinearities in trajectories of cortical myelination appeared somewhat more related to structural correlation trajectories than subtle nonlinearities in trajectories of cortical thinning.

## Discussion

In the current study we set out to examine the developmental trajectories of human brain structural networks. To this end, we used a novel “sliding window” method of network analysis to resolve age-related changes in human brain structural correlations and probabilistically thresholded brain graphs estimated from MRI data on an age-stratified sample of healthy adolescents and young adults (*N* = 297, aged 14–24 years). We found that global strength of structural correlation and the related topological property of edge density both decreased nonlinearly as a function of age: an early phase (14–19.5 years approximately) of rapid decrease in structural correlation was followed by a later phase (20–24 years) of stable or slightly increasing structural correlation. At a regional or nodal level of analysis, cortical areas varied in the magnitude of age-related decrease in nodal degree Δ*k*_max_ and the age at which nodal degree reached its minimum value age(*k*_min_). The 75 cortical areas with significantly decreasing degree tended to mature later, that is, large negative Δ*k*_max_ was associated with older age(*k*_min_). Further, cortical areas with the greatest shrinkage of degree during adolescence also had the greatest shrinkage of connection distance, that is, large negative Δ*k*_max_ was associated with large negative Δ*d*_max_. To contextualize these results, we showed that cortical areas with the greatest adolescent changes in brain structural connectivity were anatomically concentrated in regions of association cortex that had fast local rates of increasing intracortical myelination; and were topologically concentrated on the edges within frontal communities (von Economo classes 2 and 3 and the functional frontoparietal control network) and the edges connecting frontal communities to the rest of the network. We propose that these results are consistent with the existence of a developmental window for tuning of association cortical connectivity by a combination of parsimoniously pruning some long distance connections while actively consolidating or myelinating the connections which survive.

### MRI Studies of Adolescent Structural Brain Network Development

Adolescent changes in structural correlation networks have previously been investigated, as pairwise changes across four discrete (nonoverlapping) age-bins spanning the range 5–18 years (Zielinski et al. 2010; Khundrakpam et al. 2013). Zielinski et al. (2010) reported largely nonlinear changes in the extent of seed-based structural correlation networks. Both the executive control network (seeded in the right dorsolateral prefrontal cortex) and the salience network (seeded in the right frontal insula), showed an increase in spatial extent, quantified as the number of voxels whose grey matter intensity significantly correlated with the seed. Conversely, our approach suggests a decrease in the structural correlation within association areas and related structural, cytoarchitectonic, and functional communities. Beyond the difference in methods (voxel-wise seed-based vs. parcel-wise all-to-all regions), this discrepancy could be due to the different morphometric measures used, known to show differences in both trajectories of adolescent maturation (Wierenga et al. 2014; Ducharme et al. 2015), and (age-invariant) structural correlation (Sanabria-Diaz et al. 2010; Yang et al. 2016). Further, Khundrakpam et al. (2013) reported decreases in regional efficiency of primary sensorimotor regions, alongside increases in regional efficiency of paralimbic and association regions. These results align with our own, through the strong dependence of the properties of graphs thresholded to fixed edge densities (as in Khundrakpam et al. (2013)) on the mean of the correlation distributions from which they were derived. Networks with lower correlations lead to more random topology, exhibiting higher efficiency and lower clustering (Fornito et al. 2013; van den Heuvel et al. 2017). Therefore, our finding of decreases in structural correlation within association cortical areas aligns with reports by Khundrakpam et al. (2013) of increased regional efficiency in these regions. Beyond development of structural networks resolved using distinct age-groups, several studies have investigated coordinated maturation of cortical morphology during adolescence (Raznahan et al. 2011; Alexander-Bloch, Raznahan et al. 2013; Sotiras et al. 2017).

Adolescent development of structural connectivity has also been investigated using diffusion imaging and tractography, although such studies report heterogeneous findings. Lim et al. (2013) showed decreases in structural connectivity from childhood (4 years) to adulthood (40 years), concentrated predominantly on strong tracts, located within modules—which qualitatively agrees with our findings. However, Chen et al. (2013) reported increases in the number of streamlines and edge density from childhood (5 years) to adulthood (30 years). Recently, Baum et al. (2017) reported increases in within-module connectivity, and decreases in between-module connectivity in tractography-derived white matter networks. While tractography-derived structural connectomes show some overlap with structural correlation networks (Gong et al. 2012), interpretation of developmental changes in white-matter connectivity relative to development of structural correlations will require concurrent studies of both modalities in the same datasets. It is worth noting that when grey and white matter structural networks were both constructed using the same method (structural correlation), both showed similar patterns of correlation and similar developmental changes from 7 to 14 years (Moura et al. 2017).

Adolescent development of brain connectivity has also been investigated using fMRI. Early functional connectivity studies have reported increases in the strength of long-range and within-network functional connections (and decreases in the strength of short-range functional connections) (Fair et al. 2009; Supekar et al. 2009; Dosenbach et al. 2010). Later studies have reported qualitatively similar findings, but with attenuated effect sizes following control for the effects of motion (Satterthwaite et al. 2012, 2013). While findings such as increasing within-module functional connectivity may seem to disagree with our findings of decreased within-network structural correlation, these constitute disparate modalities that have not always yielded concomitant results (Fornito and Bullmore 2015). Beyond studies concurrently investigating adolescent development of structural and functional networks using the same dataset(s), the combination of structural, diffusion, and functional MRI data using methods such as multimodal fusion (Calhoun and Sui 2016), computational modeling (Breakspear 2017) or morphometric similarity (Seidlitz et al. 2017) might be useful to reconcile findings from diverse modalities.

### Relationship to Axo-synaptic Connectivity (and its Adolescent Pruning)

Our results extend previous studies of structural network development (Zielinski et al. 2010; Khundrakpam et al. 2013) by reporting smooth and nonlinear trajectories of structural network development during adolescence. The early phase of major decrease in structural correlation, nodal degree, and nodal connection distance could represent loss of anatomical connectivity to association cortical areas. The simplest interpretation is that reduced structural correlation or degree represents pruning of synaptic connections or attenuation of axonal projections. There is a large body of prior evidence in support of the concept of synaptic pruning during adolescence (Huttenlocher and Dabholkar 1997; Petanjek et al. 2011) and this mechanism has been suggested to explain age-related cortical shrinkage (Tau and Peterson 2009), which was correlated with age-related degree shrinkage in these data. However, the security of this interpretation rests on the more fundamental assumption that structural correlation measured from MRI data on multiple subjects is a reasonable proxy marker of the average weight of axo-synaptic connectivity between regions (Alexander-Bloch, Giedd et al. 2013). Beyond humans (Gong et al. 2012), there is evidence of such correspondence from animal models (Yee et al. 2017).

The identification of structural correlation networks in mice (Pagani et al. 2016) suggests that they might encompass general features of cortical architecture. Specifically, up to 35% variance in structural correlation in mice was explained by a combination of tract-tracing-derived structural connectivity, gene expression and distance (Yee et al. 2017), providing a link of the macroscopic structural networks to underlying microscale cortical organization. The relationship of structural correlation networks to gene expression has also been investigated within humans using the present data, demonstrating overlap between regional co-expression of genes (Hawrylycz et al. 2012), particularly of a subset of genes enriched in supragranular layers of cerebral cortex, and structural correlation patterns (Romero-Garcia et al. 2017). Moreover, association cortical hubs of the (age-invariant) structural correlation network showed the greatest expression of genes related to synaptic transmission, oligodendroglia as well as schizophrenia, suggesting a potential pathogenic role in abnormal consolidation of association cortical regions (Whitaker, Vértes et al. 2016). Generally, the profound adolescent maturational changes in cortical architecture are thought to underlie the frequent emergence of psychiatric disease in this period, as a result of abnormal development (Paus et al. 2008; Silbereis et al. 2016).

### Adolescent Maturation of Structural Correlation and Regional Cortical Structure

We note that the association of changes in structural network architecture to rates of cortical thinning is relatively weak. Given that (age-invariant) structural correlation networks are thought to emerge as a result of synchronized maturation (thinning) of cortical regions over adolescence (Raznahan et al. 2011; Alexander-Bloch, Raznahan et al. 2013), perhaps the changes in structural correlation might be more closely related to changes in the “rates of change” of cortical thinning, which in a longitudinal dataset were shown to peak in adolescence (Zhou et al. 2015). An additional possible explanation for the adolescent decrease in structural correlation is a “decoherence” related to interindividual differences in the timing of maturation of association areas—although the verification of such a hypothesis would again require longitudinal data. On a related note, recent work on functional connectivity has shown an adolescent increase the “distinctiveness” of individual functional connectomes (Kaufmann et al. 2017). We further note that the association of changes in structural network architecture to rates of myelination is stronger (than to rates of cortical thinning), and that subtle nonlinearities in trajectories of myelination seem more strongly related to nonlinearities in trajectories of structural correlation, suggestive of the idea that myelination may be a driver of (changes) in structural covariance. This could be further investigated through concurrent analysis of (adolescent) changes in structural correlation and white matter architecture.

Generally, the weakness of association between rates of change of morphology (ΔCT and ΔMT) and structural network architecture (Δ*k*_max_) suggests that rates of change of structural network properties explain substantial variation of brain structure with age, above and beyond the rates of thinning and myelination. As an intrinsic regional measure, cortical thickness can be considered less complex than a measure of relationships between regions (across participants) such as structural correlation; however, the biological hierarchy could well be the opposite, whereby cortical thickness and its changes might be a signature of underlying changes in axonal connectivity (which is related to structural correlation). This hypothesis could be tested, using invasive studies of concurrent development of axonal connectivity and cortical thickness in model species. In humans, the differential variance contained within cortical morphology and structural network architecture could be investigated through further within-population comparisons of these measures, in (1) their ability to discriminate between case–control populations, (2) their association to behavioral and cognitive measures, and (3) their heritability. For example, patients with childhood-onset schizophrenia have shown differences in adolescent trajectories of both cortical thinning (Alexander-Bloch et al. 2014) and structural correlation (Zalesky et al. 2015) relative to healthy controls, but the measures have not been explicitly compared.

Notably, changes in structural network architecture were more strongly related to the rate of myelination (at 70% depth) than the rate of cortical thinning, suggesting that layer-specific intracortical myelination might be a more sensitive marker of corticocortical connectivity than cortical thickness (assuming, as above, that structural correlation is a marker of connectivity). Moreover, this finding echoes our earlier finding of the rate of myelination being fastest at 70% depth between the pial surface and the grey/white matter boundary, and the relationship between rate of cortical thinning and rate of myelination being strongest at this depth (Whitaker, Vértes et al. 2016). We have previously suggested a link of these changes to histological evidence of greatest rates of myelination at similar cortical depths in rodents (Mengler et al. 2014; Tomassy et al. 2014; Hammelrath et al. 2016).

### Methodological Considerations

Recently, a number of studies have pointed out effects of participant motion on the quality of structural MRI scans, including on estimates of regional morphological measures such as cortical thickness (Reuter et al. 2015; Alexander-Bloch et al. 2016; Savalia et al. 2017). While we have carried out stringent quality control of our structural scans and FreeSurfer reconstructions of cortical thickness (details in Supplementary Information), we cannot completely rule out potential artefactual effects of motion on our results. Thus, further analysis of structural correlation development in datasets including estimates of head motion from volumetric tracking (Tisdall et al. 2012, 2016) or novel automated estimates of data quality (Shehzad et al. 2015; Pizarro et al. 2016; Rosen et al. 2017) will be important in the future.

The estimated changes in structural network organization are inevitably dependent on parameters of the sliding window method used. The selection of sliding window parameters, including window width and step size (in units of number of participants) involves several trade-offs. On one hand, selecting a wider window increases the robustness of correlations within each of those windows, as they are estimated using more participants; on the other hand, the median ages of participants within each window will cover a narrower portion of the overall age-range. Furthermore, while a smaller step size will provide a greater density of windows and hence time-points for curve fitting and trajectory characterization, a denser sampling of data will exacerbate issues with the inevitably uneven distribution of subjects across the age-range studied, which in effect corresponds to an unevenly sampled time-series. Future development of tools for the analysis of unevenly sampled time-series (Eckner 2014) should help alleviate these issues.

Furthermore, depending on the sliding window parameters, relatively few summary data points may be obtained. The subsequent fitting of nonlinear smoothing splines (with up to ~3.5 degrees of freedom) to such scarce data warrants care when interpreting evidence of nonlinearity—despite evidence from both the AIC and BIC that smoothing splines provide a better quality of fit than linear models. Still, it is reassuring that trajectories remain consistently nonlinear across bootstrapped samples (within unthresholded correlation networks) and that evidence of a nonlinear trajectory seems more pronounced after bootstrap thresholding. Changes in structural network architecture remain qualitatively consistent in both their spatial location and relationship to changes in morphology when simple, linear models are used. The scarcity of data points may also lead to uncertainties in measures used to characterize the maturational trajectories, including the measures of maximum change and age at minimum of the trajectory. Finally, it remains ambiguous whether the tendency of the global trajectory of structural correlation to slightly increase from the minimum around age 19 towards age 24 years is significant, or whether the trajectory can be seen as levelling-off. It seems reasonable that the few nodes presenting increases in structural correlation (e.g., within right cingulate cortex) would be driving this effect. Thus, until these results are validated in an additional dataset, care is necessary in some aspects of their interpretation.

Further, practical applicability of structural correlation networks is limited by the fact that they represent a group construct. Still, an advantage of structural correlation networks over structural connectomes derived from diffusion imaging using tractography is the relative simplicity of the structural MRI acquisitions compared with diffusion imaging, which in light of its longer acquisition is more prone to motion artefacts (Yendiki et al. 2014), and within which tractography presents considerable challenges (Thomas et al. 2014; Reveley et al. 2015; Maier-Hein et al. 2016). Efforts to derive measures of individual contribution to structural correlation networks (Saggar et al. 2015) or fully individual networks from structural imaging (Tijms et al. 2012; Kong et al. 2014, 2015) including through the combination of multimodal features (Seidlitz et al. 2017) should increase the practical applicability of structural correlation network research.

In reporting a late maturation of association cortical regions, our results are potentially compatible with the developmental mismatch hypothesis, which proposes that late maturation of prefrontal regions (involved in cognitive control), compared with an earlier development of subcortical regions (implicated in reward processing) results in adolescent increases in risk-taking and sensation-seeking behaviors (Mills et al. 2014). However, the verification of such a hypothesis will require the inclusion of both subcortical regions and behavioral data in future analyses.

Finally, structural network architecture is known to mature across the lifespan (DuPre and Spreng 2017), including during both early childhood (Geng et al. 2017) and late adulthood (Hafkemeijer et al. 2014). Our focused age-range prohibits us from conclusively ascertaining the specificity of these changes to adolescence. For example, extending the analyses presented herein to wider age-ranges would help disambiguate whether the nonlinear decreases in structural correlation level off or increase in young adulthood. In general, the wide applicability of the methods used herein should enable investigations of the maturation of structural brain networks, as well as other networks constructed in a similar manner (including for example networks of relationships between psychopathological symptoms; Borsboom and Cramer 2013), across the lifespan.

## Conclusion

During adolescence, human brain structural correlation networks demonstrate a nonlinear reduction of connectivity of association cortical areas, predominantly in frontal cortex, that is compatible with a developmental process of pruning combined with consolidation of surviving connections.

## Availability of Data and Code

Data for this specific article has been uploaded to the Cambridge Data Repository (https://doi.org/10.17863/CAM.8856) and password protected. Our participants did not give informed consent for their questionnaire measures to be made publicly available, and it is possible that they could be identified from this data set. Access to the data supporting the analyses presented in this article will be made available to researchers with a reasonable request to NSPNdata@medschl.cam.ac.uk. The code used to conduct analyses is available from F.V.’s github: https://github.com/frantisekvasa/structural_network_development (DOI: 10.5281/zenodo.528674).

## Supplementary Material

Supplementary Data

## References

[bhx249C1] Alexander-Bloch A, Clasen L, Stockman M, Ronan L, Lalonde F, Giedd J, Raznahan A 2016 Subtle in-scanner motion biases automated measurement of brain anatomy from in vivo MRI. Hum Brain Mapp. 37:2385–2397. 27004471 10.1002/hbm.23180 PMC5110234

[bhx249C2] Alexander-Bloch A, Giedd JN, Bullmore E 2013 Imaging structural co-variance between human brain regions. Nat Rev Neurosci. 14:322–336. 23531697 10.1038/nrn3465 PMC4043276

[bhx249C3] Alexander-Bloch A, Raznahan A, Bullmore E, Giedd J 2013 The convergence of maturational change and structural covariance in human cortical networks. J Neurosci. 33:2889–2899. 23407947 10.1523/JNEUROSCI.3554-12.2013 PMC3711653

[bhx249C4] Alexander-Bloch A, Reiss PT, Rapoport J, Mcadams H, Giedd JN, Bullmore ET, Gogtay N 2014 Abnormal cortical growth in schizophrenia targets normative modules of synchronized development. Biol Psychiatry. 76:438–446. 24690112 10.1016/j.biopsych.2014.02.010 PMC4395469

[bhx249C5] Baum GL, Ciric R, Roalf DR, Betzel RF, Moore TM, Shinohara RT, Kahn AE, Vandekar SN, Rupert PE, Quarmley M, et al 2017 Modular segregation of structural brain networks supports the development of executive function in youth. Curr Biol. 27:1561–1572.e8. 28552358 10.1016/j.cub.2017.04.051 PMC5491213

[bhx249C6] Blondel VD, Guillaume J-L, Lambiotte R, Lefebvre E 2008 Fast unfolding of communities in large networks. J Stat Mech Theory Exp. 10008:6.

[bhx249C7] Borsboom D, Cramer AOJ 2013 Network analysis: an integrative approach to the structure of psychopathology. Annu Rev Clin Psychol. 9:91–121. 23537483 10.1146/annurev-clinpsy-050212-185608

[bhx249C8] Breakspear M 2017 Dynamic models of large-scale brain activity. Nat Neurosci. 20:340–352. 28230845 10.1038/nn.4497

[bhx249C9] Bullmore E, Sporns O 2009 Complex brain networks: graph theoretical analysis of structural and functional systems. Nat Rev Neurosci. 10:186–198. 19190637 10.1038/nrn2575

[bhx249C10] Calhoun VD, Sui J 2016 Multimodal fusion of brain imaging data: a key to finding the missing link(s) in complex mental illness. Biol Psychiatry Cogn Neurosci Neuroimaging. 1:230–244. 27347565 10.1016/j.bpsc.2015.12.005 PMC4917230

[bhx249C11] Chen Z, Liu M, Gross DW, Beaulieu C 2013 Graph theoretical analysis of developmental patterns of the white matter network. Front Hum Neurosci. 7:716. 24198774 10.3389/fnhum.2013.00716 PMC3814848

[bhx249C12] Desikan RS, Ségonne F, Fischl B, Quinn BT, Dickerson BC, Blacker D, Buckner RL, Dale AM, Maguire RP, Hyman BT, et al 2006 An automated labeling system for subdividing the human cerebral cortex on MRI scans into gyral based regions of interest. Neuroimage. 31:968–980. 16530430 10.1016/j.neuroimage.2006.01.021

[bhx249C13] Dosenbach NUF, Nardos B, Cohen AL, Fair DA, Power JD, Church JA, Nelson SM, Wig GS, Vogel AC, Lessov-Schlaggar CN, et al 2010 Prediction of individual brain maturity using fMRI. Science. 329:1358–1361. 20829489 10.1126/science.1194144 PMC3135376

[bhx249C14] Ducharme S, Albaugh MD, Nguyen TV, Hudziak JJ, Mateos-Pérez JM, Labbe A, Evans AC, Karama S, Brain Development Cooperative Group 2015 Trajectories of cortical surface area and cortical volume maturation in normal brain development. Data Brief. 5:929–938. 26702424 10.1016/j.dib.2015.10.044 PMC4669480

[bhx249C15] DuPre E, Spreng RN 2017 Structural covariance networks across the lifespan, from 6–94 years of age. Netw Neurosci. 1–38. http://www.mitpressjournals.org/doi/abs/10.1162/NETN_a_00016. 29855624 10.1162/NETN_a_00016 PMC5874135

[bhx249C16] Eckner A (2014). Some properties of operators for unevenly spaced time series. Available from: URL http://www.eckner.com/research.html.

[bhx249C17] Evans AC 2013 Networks of anatomical covariance. Neuroimage. 80:489–504. 23711536 10.1016/j.neuroimage.2013.05.054

[bhx249C18] Fair DA, Cohen AL, Power JD, Dosenbach NUF, Church JA, Miezin FM, Schlaggar BL, Petersen SE 2009 Functional brain networks develop from a “local to distributed” organization. PLoS Comput Biol. 5:e1000381. 19412534 10.1371/journal.pcbi.1000381 PMC2671306

[bhx249C19] Fischl B, Sereno MI, Dale AM 1999 Cortical surface-based analysis. II: Inflation, flattening, and a surface-based coordinate system. Neuroimage. 9:195–207. 9931269 10.1006/nimg.1998.0396

[bhx249C20] Fjell AM, Walhovd KB, Westlye LT, Østby Y, Tamnes CK, Jernigan TL, Gamst A, Dale AM 2010 When does brain aging accelerate? Dangers of quadratic fits in cross-sectional studies. Neuroimage. 50:1376–1383. 20109562 10.1016/j.neuroimage.2010.01.061

[bhx249C21] Fornito A, Bullmore ET 2015 Reconciling abnormalities of brain network structure and function in schizophrenia. Curr Opin Neurobiol. 30:44–50. 25238608 10.1016/j.conb.2014.08.006

[bhx249C22] Fornito A, Zalesky A, Breakspear M 2013 Graph analysis of the human connectome: Promise, progress, and pitfalls. Neuroimage. 80:426–444. 23643999 10.1016/j.neuroimage.2013.04.087

[bhx249C23] Fornito A, Zalesky A, Bullmore ET 2016 Fundamentals of brain network analysis. 1st ed. Cambridge, MA: Academic Press.

[bhx249C24] Geng X, Li G, Lu Z, Gao W, Wang L, Shen D, Zhu D, Gilmore JH 2017 Structural and maturational covariance in early childhood brain development. Cereb Cortex. 27(3):1795–1807. 26874184 10.1093/cercor/bhw022 PMC6059236

[bhx249C25] Gong G, He Y, Chen ZJ, Evans AC 2012 Convergence and divergence of thickness correlations with diffusion connections across the human cerebral cortex. Neuroimage. 59:1239–1248. 21884805 10.1016/j.neuroimage.2011.08.017

[bhx249C26] Hafkemeijer A, Altmann-Schneider I, de Craen AJM, Slagboom PE, van der Grond J, Rombouts SARB 2014 Associations between age and gray matter volume in anatomical brain networks in middle-aged to older adults. Aging Cell. 13:1068–1074. 25257192 10.1111/acel.12271 PMC4326918

[bhx249C27] Hammelrath L, Škokić S, Khmelinskii A, Hess A, van der Knaap N, Staring M, Lelieveldt BPF, Wiedermann D, Hoehn M 2016 Morphological maturation of the mouse brain: an in vivo MRI and histology investigation. Neuroimage. 125:144–152. 26458518 10.1016/j.neuroimage.2015.10.009

[bhx249C28] Hawrylycz MJ, Lein ES, Guillozet-Bongaarts AL, Shen EH, Ng L, Miller JA, van de Lagemaat LN, Smith KA, Ebbert A, Riley ZL, et al 2012 An anatomically comprehensive atlas of the adult human brain transcriptome. Nature. 489:391–399. 22996553 10.1038/nature11405 PMC4243026

[bhx249C29] Huttenlocher PR, Dabholkar AS 1997 Regional differences in synaptogenesis in human cerebral cortex. J Comp Neurol. 387:167–178. 9336221 10.1002/(sici)1096-9861(19971020)387:2<167::aid-cne1>3.0.co;2-z

[bhx249C30] Kaufmann T, Alnæs D, Doan NT, Brandt CL, Andreassen OA, Westlye LT 2017 Delayed stabilization and individualization in connectome development are related to psychiatric disorders. Nat Neurosci. 10.1038/nn.4511. 28218917

[bhx249C31] Khundrakpam BS, Reid A, Brauer J, Carbonell F, Lewis J, Ameis S, Karama S, Lee J, Chen Z, Das S, et al 2013 Developmental changes in organization of structural brain networks. Cereb Cortex. 23:2072–2085. 22784607 10.1093/cercor/bhs187 PMC3729193

[bhx249C32] Kong XZ, Liu Z, Huang L, Wang X, Yang Z, Zhou G, Zhen Z, Liu J 2015 Mapping individual brain networks using statistical similarity in regional morphology from MRI. PLoS One. 10:1–24. 10.1371/journal.pone.0141840 PMC4633111 26536598

[bhx249C33] Kong XZ, Wang X, Huang L, Pu Y, Yang Z, Dang X, Zhen Z, Liu J 2014 Measuring individual morphological relationship of cortical regions. J Neurosci Methods. 237:103–107. 25220868 10.1016/j.jneumeth.2014.09.003

[bhx249C34] Lancichinetti A, Fortunato S 2012 Consensus clustering in complex networks. Sci Rep. 2 10.1038/srep00336. PMC3313482 22468223

[bhx249C35] Lerch JP, Worsley K, Shaw WP, Greenstein DK, Lenroot RK, Giedd J, Evans AC 2006 Mapping anatomical correlations across cerebral cortex (MACACC) using cortical thickness from MRI. Neuroimage. 31:993–1003. 16624590 10.1016/j.neuroimage.2006.01.042

[bhx249C36] Lim S, Han CE, Uhlhaas PJ, Kaiser M 2013 Preferential detachment during human brain development: age- and sex-specific structural connectivity in diffusion tensor imaging (DTI) data. Cereb Cortex. 25:1–13. 24343892 10.1093/cercor/bht333 PMC4428296

[bhx249C37] Maier-Hein K, Neher P, Houde J-C, Cote M-A, Garyfallidis E, Zhong J, Chamberland M, Yeh F-C, Lin YC, Ji Q, et al 2016 Tractography-based connectomes are dominated by false-positive connections. bioRxiv. 1–23.

[bhx249C38] Mengler L, Khmelinskii A, Diedenhofen M, Po C, Staring M, Lelieveldt BPF, Hoehn M 2014 Brain maturation of the adolescent rat cortex and striatum: changes in volume and myelination. Neuroimage. 84:35–44. 23994458 10.1016/j.neuroimage.2013.08.034

[bhx249C39] Miller DJ, Duka T, Stimpson CD, Schapiro SJ, Baze WB, McArthur MJ, Fobbs AJ, Sousa AM, Sestan N, Wildman DE, et al 2012 Prolonged myelination in human neocortical evolution. Proc Natl Acad Sci USA. 109:16480–16485. 23012402 10.1073/pnas.1117943109 PMC3478650

[bhx249C40] Mills KL, Goddings A-L, Clasen LS, Giedd JN, Blakemore S-J 2014 The developmental mismatch in structural brain maturation during adolescence. Dev Neurosci. 36:147–160. 24993606 10.1159/000362328

[bhx249C41] Moura LM, Crossley NA, Zugman A, Pan PM, Gadelha A, Del Aquilla MAG, Picon FA, Anés M, Amaro E Jr, de Jesus Mari J, et al 2017 Coordinated brain development: exploring the synchrony between changes in grey and white matter during childhood maturation. Brain Imaging Behav. 11:808–817. 27169540 10.1007/s11682-016-9555-0

[bhx249C42] Pagani M, Bifone A, Gozzi A 2016 Structural covariance networks in the mouse brain. Neuroimage. 129:55–63. 26802512 10.1016/j.neuroimage.2016.01.025

[bhx249C43] Paus T, Keshavan M, Giedd JN 2008 Why do many psychiatric disorders emerge during adolescence? Nat Rev Neurosci. 9:947–957. 19002191 10.1038/nrn2513 PMC2762785

[bhx249C44] Petanjek Z, Judas M, Simic G, Rasin MR, Uylings HBM, Rakic P, Kostovic I 2011 Extraordinary neoteny of synaptic spines in the human prefrontal cortex. Proc Natl Acad Sci USA. 108:13281–13286. 21788513 10.1073/pnas.1105108108 PMC3156171

[bhx249C45] Pizarro RA, Cheng X, Barnett A, Lemaitre H, Verchinski BA, Goldman AL, Xiao E, Luo Q, Berman KF, Callicott JH, et al 2016 Automated quality assessment of structural magnetic resonance brain images based on a supervised machine learning algorithm. Front Neuroinform. 10:52. 28066227 10.3389/fninf.2016.00052 PMC5165041

[bhx249C46] Raznahan A, Lerch JP, Lee N, Greenstein D, Wallace GL, Stockman M, Clasen L, Shaw PW, Giedd JN 2011 Patterns of coordinated anatomical change in human cortical development: a longitudinal neuroimaging study of maturational coupling. Neuron. 72:873–884. 22153381 10.1016/j.neuron.2011.09.028 PMC4870892

[bhx249C47] Reiss PT, Huang L, Chen Y-H, Huo L, Tarpey T, Mennes M 2014 Massively parallel nonparametric regression, with an application to developmental brain mapping. J Comput Graph Stat. 23:232–248. 24683303 10.1080/10618600.2012.733549 PMC3964810

[bhx249C48] Reuter M, Tisdall MD, Qureshi A, Buckner RL, van der Kouwe AJW, Fischl B 2015 Head motion during MRI acquisition reduces gray matter volume and thickness estimates. Neuroimage. 107:107–115. 25498430 10.1016/j.neuroimage.2014.12.006 PMC4300248

[bhx249C49] Reveley C, Seth AK, Pierpaoli C, Silva AC, Yu D, Saunders RC, Leopold DA, Ye FQ 2015 Superficial white matter fiber systems impede detection of long-range cortical connections in diffusion MR tractography. Proc Natl Acad Sci USA. 112:E2820–E2828. 25964365 10.1073/pnas.1418198112 PMC4450402

[bhx249C50] Romero-Garcia R, Atienza M, Clemmensen LH, Cantero JL 2012 Effects of network resolution on topological properties of human neocortex. Neuroimage. 59:3522–3532. 22094643 10.1016/j.neuroimage.2011.10.086

[bhx249C51] Romero-Garcia R, Whitaker KJ, Váša F, Seidlitz J, Fonagy P, Dolan RJ, Jones PB, Goodyer IM, the NSPN Consortium, et al, 2017 Structural covariance networks are coupled to expression of genes enriched in supragranular layers of the human cortex. bioRxiv, 1–25. 10.1016/j.neuroimage.2017.12.060 PMC5883331 29274746

[bhx249C52] Rosen A, Roalf DR, Ruparel K, Blake J, Seelaus K, Villa P, Cook AP, Davatzikos C, Elliott MA, de La Garza AG, et al 2017 Data-driven Assessment of Structural Image Quality. bioRxiv. 1–35.

[bhx249C53] Saggar M, Hosseini SMH, Bruno JL, Quintin E, Raman MM, Kesler SR, Reiss AL 2015 Estimating individual contribution from group-based structural correlation networks. Neuroimage. 120:274–284. 26162553 10.1016/j.neuroimage.2015.07.006 PMC4589508

[bhx249C54] Sanabria-Diaz G, Melie-García L, Iturria-Medina Y, Alemán-Gómez Y, Hernández-González G, Valdés-Urrutia L, Galán L, Valdés-Sosa P 2010 Surface area and cortical thickness descriptors reveal different attributes of the structural human brain networks. Neuroimage. 50:1497–1510. 20083210 10.1016/j.neuroimage.2010.01.028

[bhx249C55] Satterthwaite TD, Wolf DH, Loughead J, Ruparel K, Elliott MA, Hakonarson H, Gur RC, Gur RE 2012 Impact of in-scanner head motion on multiple measures of functional connectivity: relevance for studies of neurodevelopment in youth. Neuroimage. 60:623–632. 22233733 10.1016/j.neuroimage.2011.12.063 PMC3746318

[bhx249C56] Satterthwaite TD, Wolf DH, Ruparel K, Erus G, Elliott MA, Eickhoff SB, Gennatas ED, Jackson C, Prabhakaran K, Smith A, et al 2013 Heterogeneous impact of motion on fundamental patterns of developmental changes in functional connectivity during youth. Neuroimage. 83:45–57. 23792981 10.1016/j.neuroimage.2013.06.045 PMC3874413

[bhx249C57] Savalia NK, Agres PF, Chan MY, Feczko EJ, Kennedy KM, Wig GS 2017 Motion-related artifacts in structural brain images revealed with independent estimates of in-scanner head motion. Hum Brain Mapp. 38:472–492. 27634551 10.1002/hbm.23397 PMC5217095

[bhx249C58] Seidlitz J, Váša F, Shinn M, Romero-Garcia R, Whitaker KJ, Vértes PE, Reardon PK, Clasen L, Messinger A, Leopold DA, et al 2017 Morphometric Similarity Networks Detect Microscale Cortical Organisation and Predict Inter-Individual Cognitive Variation. bioRxiv. 1–63. 10.1016/j.neuron.2017.11.039 PMC5763517 29276055

[bhx249C59] Shehzad Z, Giavasis S, Li Q, Benhajali Y, Yan C, Yang Z, Milham M, Bellec P, Craddock C 2015 The preprocessed connectomes project quality assessment protocol—a resource for measuring the quality of MRI data. Front Neurosci. 9 10.3389/conf.fnins.2015.91.00047.

[bhx249C60] Shinn M, Romero-Garcia R, Seidlitz J, Váša F, Vértes PE, Bullmore E 2017 Versatility of nodal affiliation to communities. Sci Rep. 7 10.1038/s41598-017-03394-5. PMC5487331 28655911

[bhx249C61] Silbereis JC, Pochareddy S, Zhu Y, Li M, Sestan N 2016 The cellular and molecular landscapes of the developing human central nervous system. Neuron. 89:268. 10.1016/j.neuron.2015.12.008 PMC4959909 26796689

[bhx249C62] Sotiras A, Toledo JB, Gur RE, Gur RC, Satterthwaite TD, Davatzikos C 2017 Patterns of coordinated cortical remodeling during adolescence: associations with functional specialization and evolutionary expansion. Proc Natl Acad Sci USA. 10.1073/pnas.1620928114. PMC5380071 28289224

[bhx249C63] Sporns O, Betzel RF 2016 Modular brain networks. Annu Rev Psychol. 67:613–640. 26393868 10.1146/annurev-psych-122414-033634 PMC4782188

[bhx249C64] Supekar K, Musen M, Menon V 2009 Development of large-scale functional brain networks in children. PLoS Biol. 7:e1000157. 19621066 10.1371/journal.pbio.1000157 PMC2705656

[bhx249C65] Tau GZ, Peterson BS 2009 Normal development of brain circuits. Neuropsychopharmacology. 35:147–168. 10.1038/npp.2009.115 PMC3055433 19794405

[bhx249C66] Thomas C, Ye FQ, Irfanoglu MO, Modi P, Saleem KS, Leopold DA, Pierpaoli C 2014 Anatomical accuracy of brain connections derived from diffusion MRI tractography is inherently limited. Proc Natl Acad Sci USA. 111 10.1073/pnas.1405672111. PMC4246325 25368179

[bhx249C67] Tijms BM, Seriès P, Willshaw DJ, Lawrie SM 2012 Similarity-based extraction of individual networks from gray matter MRI scans. Cereb Cortex. 22:1530–1541. 21878484 10.1093/cercor/bhr221

[bhx249C68] Tisdall MD, Hess AT, Reuter M, Meintjes EM, Fischl B, van der Kouwe AJW 2012 Volumetric navigators for prospective motion correction and selective reacquisition in neuroanatomical MRI. Magn Reson Med. 68:389–399. 22213578 10.1002/mrm.23228 PMC3320676

[bhx249C69] Tisdall MD, Reuter M, Qureshi A, Buckner RL, Fischl B, van der Kouwe AJW 2016 Prospective motion correction with volumetric navigators (vNavs) reduces the bias and variance in brain morphometry induced by subject motion. Neuroimage. 127:11–22. 26654788 10.1016/j.neuroimage.2015.11.054 PMC4754677

[bhx249C70] Tomassy GS, Berger DR, Chen H-H, Kasthuri N, Hayworth KJ, Vercelli A, Seung HS, Lichtman JW, Arlotta P 2014 Distinct profiles of myelin distribution along single axons of pyramidal neurons in the neocortex. Science. 344:319–324. 24744380 10.1126/science.1249766 PMC4122120

[bhx249C71] van den Heuvel MP, de Lange SC, Zalesky A, Seguin C, Yeo BTT, Schmidt R 2017 Proportional thresholding in resting-state fMRI functional connectivity networks and consequences for patient-control connectome studies: issues and recommendations. Neuroimage. 152:437–449. 28167349 10.1016/j.neuroimage.2017.02.005

[bhx249C72] van Wijk BCM, Stam CJ, Daffertshofer A 2010 Comparing brain networks of different size and connectivity density using graph theory. PLoS One. 5:e13701. 21060892 10.1371/journal.pone.0013701 PMC2965659

[bhx249C73] Vandekar SN, Shinohara RT, Raznahan A, Roalf DR, Ross M, DeLeo N, Ruparel K, Verma R, Wolf DH, Gur RC, et al 2015 Topologically dissociable patterns of development of the human cerebral cortex. J Neurosci. 35:599–609. 25589754 10.1523/JNEUROSCI.3628-14.2015 PMC4293413

[bhx249C74] von Economo C, Koskinas GN 1925 Die Cytoarchitektonik der Hirnrinde des Erwachsenen Menschen: Textband und Atlas mit 112 Mikrophotographischen Tafeln. Vienna: Springer.

[bhx249C75] Weiskopf N, Suckling J, Williams G, Correia MM, Inkster B, Tait R, Ooi C, Bullmore ET, Lutti A 2013 Quantitative multi-parameter mapping of R1, PD(*), MT, and R2(*) at 3T: a multi-center validation. Front Neurosci. 7:95. 23772204 10.3389/fnins.2013.00095 PMC3677134

[bhx249C76] Whitaker KJ, Vértes PE, Romero-Garcia R, Váša F, Moutoussis M, Prabhu G, Weiskopf N, Callaghan MF, Wagstyl K, Rittman T, et al 2016 Adolescence is associated with transcriptionally patterned consolidation of the hubs of the human brain connectome. Proc Natl Acad Sci USA. 113:9105–9110. 27457931 10.1073/pnas.1601745113 PMC4987797

[bhx249C77] Wierenga LM, Langen M, Oranje B, Durston S 2014 Unique developmental trajectories of cortical thickness and surface area. Neuroimage. 87:120–126. 24246495 10.1016/j.neuroimage.2013.11.010

[bhx249C78] Yang J-J, Kwon H, Lee J-M 2016 Complementary characteristics of correlation patterns in morphometric correlation networks of cortical thickness, surface area, and gray matter volume. Sci Rep. 6:1–9. 27226000 10.1038/srep26682 PMC4881013

[bhx249C79] Yee Y, Fernandes DJ, French L, Ellegood J, Cahill LS, Vousden DA, Noakes LS, Scholz J, van Eede MC, Nieman BJ, et al 2017 Structural covariance of brain region volumes is associated with both structural connectivity and transcriptomic similarity. bioRxiv. 10.1101/183004. 29782994

[bhx249C80] Yendiki A, Koldewyn K, Kakunoori S, Kanwisher N, Fischl B 2014 Spurious group differences due to head motion in a diffusion MRI study. Neuroimage. 88:79–90. 24269273 10.1016/j.neuroimage.2013.11.027 PMC4029882

[bhx249C81] Yeo B, Krienen F 2011 The organization of the human cerebral cortex estimated by intrinsic functional connectivity. J Neurophysiol. 106:1125–1165. 21653723 10.1152/jn.00338.2011 PMC3174820

[bhx249C100] Yeo BTT, Krienen FM, Sepulcre J, Sabuncu MR, Lashkari D, Hollinshead M … Buckner RL 2011 The organization of the human cerebral cortex estimated by intrinsic functional connectivity. J Neurophysiol. 106(3):1125–1165. 21653723 10.1152/jn.00338.2011 PMC3174820

[bhx249C82] Zalesky A, Pantelis C, Cropley V, Fornito A, Cocchi L, McAdams H, Clasen L, Greenstein D, Rapoport JL, Gogtay N 2015 Delayed development of brain connectivity in adolescents with schizophrenia and their unaffected siblings. JAMA Psychiatry. 72:900–908. 26176706 10.1001/jamapsychiatry.2015.0226

[bhx249C83] Zhou D, Lebel C, Treit S, Evans A, Beaulieu C 2015 Accelerated longitudinal cortical thinning in adolescence. Neuroimage. 104:138–145. 25312772 10.1016/j.neuroimage.2014.10.005

[bhx249C84] Zielinski B. a, Gennatas ED, Zhou J, Seeley WW 2010 Network-level structural covariance in the developing brain. Proc Natl Acad Sci USA. 107:18191–18196. 20921389 10.1073/pnas.1003109107 PMC2964249

