# Supplementary material for: Adolescent Tuning of Association Cortex in Human Structural Brain Networks
Source: Cereb Cortex. 2017 Oct 27;28(1):281–94. doi: 10.1093/cercor/bhx249 (PMC5903415; doi:10.1093/cercor/bhx249)
Supplement: Supplementary Data [file bhx249_supplements.zip › vasa_et_al_Cerebral_Cortex_Supplementary_Information.pdf]

Supplementary Information for:
Adolescent tuning of association cortex in human structural brain networks

František Váša[1], Jakob Seidlitz[1,2], Rafael Romero-Garcia[1], Kirstie J. Whitaker[1,3], Gideon Rosenthal[4], Petra E. Vértes[1], Maxwell Shinn[1], Aaron Alexander-Bloch[5], Peter Fonagy[6], Raymond J. Dolan[7,8], Peter B. Jones[1,9], Ian M. Goodyer[1,9], the NSPN consortium[#], Olaf Sporns[10], Edward T. Bullmore[1,9,11]

[1] Brain Mapping Unit, Department of Psychiatry, University of Cambridge, Cambridge, CB2 0SZ, UK
[2] Developmental Neurogenomics Unit, National Institute of Mental Health, Bethesda, MD 20892, USA
[3] The Alan Turing Institute for Data Science, British Library, London, NW1 2DB, UK
[4] Department of Brain and Cognitive Sciences, Ben-Gurion University of the Negev, PO Box 653, Beer-Sheva 8410501, Israel
[5] Department of Psychiatry, Yale University School of Medicine, New Haven, CT 06511, USA
[6] Research Department of Clinical, Educational and Health Psychology, University College London, London, WC1E 6BT, UK
[7] Wellcome Trust Centre for Neuroimaging, UCL Institute of Neurology, University College London, London, WC1N 3BG, UK
[8] Max Planck University College London Centre for Computational Psychiatry and Ageing Research, University College London, London, WC1B 5EH, United Kingdom
[9] Cambridgeshire & Peterborough NHS Foundation Trust, Huntingdon, PE29 3RJ, UK
[10] Department of Psychological and Brain Sciences, Indiana University, Bloomington, IN 47405, USA
[11] Immunology & Inflammation Therapeutic Area Unit, GlaxoSmithKline R&D, Stevenage, SG1 2NY, UK

[#] List of NSPN consortium members on p. 30

Contents

Details of data acquisition and processing..p. 3
Quality control.. p. 3
Assignment of nodes to communities.. p. 3
Module decomposition of the age-invariant structural network.................. p. 3
The von Economo atlas of cytoarchitectonic classes.................................... p. 6
Functional intrinsic connectivity networks.. p. 6
Spatial permutation test...p. 7
Trajectories of structural correlation, covariance and variance................................ p. 9
Unthresholded trajectories of node degree ....p. 11
Unthresholded regional trajectories of correlation strength....................................p. 12
Trajectories of Euclidean distance.. p. 13
Rates of thinning and myelination.. p. 14
Node degree within the age-invariant structural correlation network..................... p. 15
Linear rates of change of node degree...p. 16
Sensitivity Analyses.. p. 17
(In)dependence of results on sliding window parameters............................ p. 17
Effects of gender...p. 21
Effects of regional reliability...p. 21
Effects of irregularities in age-distribution of participants.............................p. 23
Effects of non-linearities in trajectories of cortical thinning & myelination p. 25
Nonlinearities in cortical thickness.. p. 25
Nonlinearities in myelination..p. 26
Sub-sample analysis.. p. 28
Neuroscience in Psychiatry Network (NSPN) Consortium author list........................p. 30
Supplementary References..p. 31

Details of data acquisition and processing

The following details concerning the multi-parametric mapping (MPM) sequence have previously been described by Whitaker, Vértes et al. (2016) and Weiskopf et al. (2013).

The MPM sequence comprised three multi-echo 3D FLASH (fast low angle shot) scans, with predominant weighting determined by choice of the repetition time (TR) and the flip angle α: for the T1 scan, TR = 18.7 ms, α = 20°; for the MT scan, TR = 23.7 ms, α = 6°. Multiple gradient echoes were acquired with alternating readout polarity at six equidistant echo times (TE) between 2.2 and 14.7 ms for both acquisitions. Other acquisition parameters were: 1 mm isotropic resolution, 176 sagittal partitions, field of view (FOV) = 256 × 240 mm, matrix = 256 × 240 ×176, parallel imaging using GRAPPA factor 2 in phase-encoding (PE) direction (AP), 6/8 partial Fourier in partition direction, non-selective RF excitation, readout bandwidth BW = 425 Hz/pixel, RF spoiling phase increment = 50°. The total acquisition time was ~25 min. Participants were given standard ear protection and instructed to lie still and rest during the scan.

Quality control

All FreeSurfer reconstructions were visually inspected and manually edited by members of the NSPN Consortium (KJW, RRG, FV and Konrad Wagstyl). The recon-all algorithm was run after each round of manual quality control and up to 10 iterations of edits were performed. Complete cortical reconstruction was required for data to be included in the final analysis. There were 320 scans collected with 23 scans excluded as they did not meet these quality requirements.

Assignment of nodes to communities

Module decomposition of the age-invariant structural correlation network

The community structure of the bootstrap-thresholded (but weighted) age-invariant structural correlation network was decomposed using the Louvain multi-resolution algorithm. As the resolution parameter γ is increased, the community structure is decomposed into a progressively larger number of modules. We identified values of the resolution parameter which reduce the mean versatility, a measure of the uncertainty with which a node is assigned to a module (Shinn et al., 2017). The mean versatility does not provide an objective global optimum of the resolution parameter γ; instead, it serves to guide optimisation of γ to local minima within neighbourhoods corresponding to the desired spatial resolution of the modules. We estimated mean versatility across 200 runs of the Louvain algorithm for each value of γ in the range $0.01 \leq \gamma \leq 4.00$, incremented in steps of 0.01. Five local minima of mean versatility were identified (γ = 0.94, 1.06, 1.12, 1.19, 1.30; **SI Fig. S1A**). Solutions of $\gamma < 0.85$ (where μ versatility = 0) yielded a trivial solution of a single module; local minima in the upper range of examined γ values yielded too many modules to be practical (e.g.: 233 modules for γ = 2.44). The modular architecture corresponding to each local minimum of mean versatility was obtained as a consensus partition (Lancichinetti & Fortunato, 2012) across 1000 runs of the Louvain modularity algorithm using the corresponding value of γ (**Fig. S1B**). For further

analyses of the development of intra-and inter-modular structural network architecture, we chose the partition which divided the network into seven modules (γ = 1.19), for ease of comparison to the equally numerous cytoarchitectonic classes and functional networks. For details of node assignments to modules, see **Fig. S1Biv** and **Table S1**. Analyses of changes in structural network architecture using other modular partitions (from the other local minima of γ) were qualitatively consistent (results not shown).

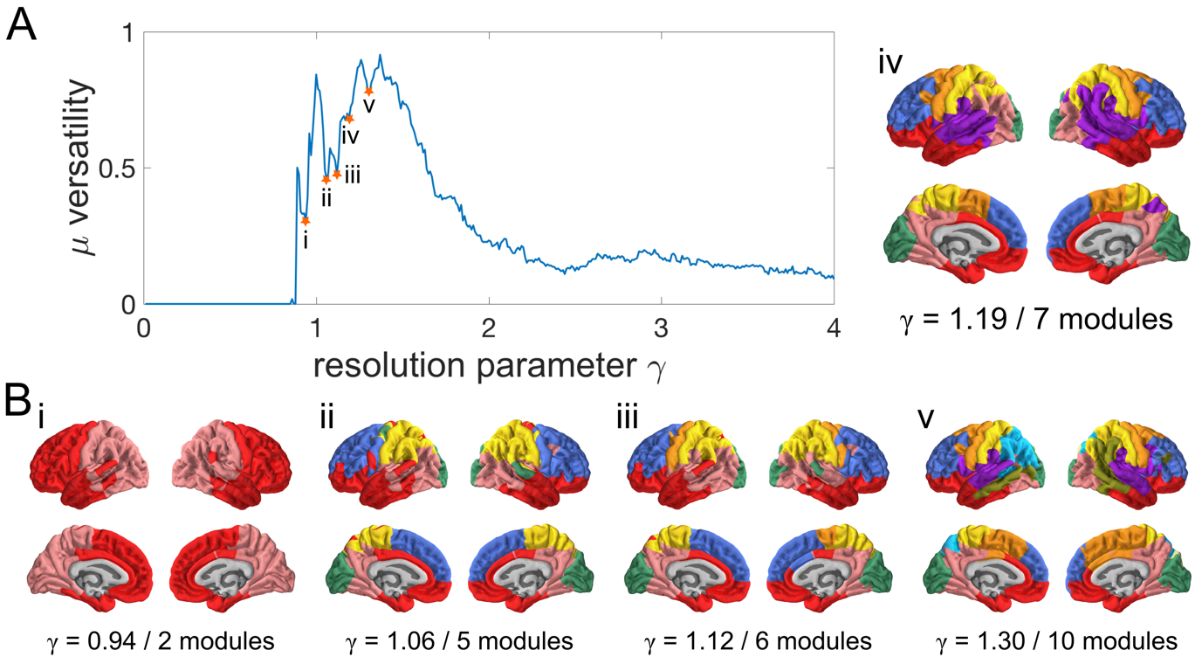

Supplementary Figure S1: Partitions of the age-invariant structural correlation network which minimise uncertainty of node affiliation to modules. Module partitions were obtained for values of the resolution parameter γ corresponding to minima of mean versatility, a measure of the uncertainty with which a node is consistently affiliated to a module (Shinn et al., 2017). A) Mean versatility was calculated across 200 runs of the Louvain community algorithm for γ = 0.01, 0.02, …, 4.00. Five local minima were selected. B) Community organisation for the five local minima indicated on panel A, using a consensus formulation of the Louvain algorithm (Lancichinetti & Fortunato, 2012) run 1000 times at each local minimum. Partition iv was chosen due to its 7-module solution, which facilitates comparison to the equally numerous cytoarchitectonic classes and functional networks.

**module 1 (red)**	**module 2 (blue)**	**module 4 (pink)**	**module 6 (purple)**
caud. ant. cingulate	caud. mid. frontal	fusiform	banks sts
entorhinal	pars opercularis	inf. parietal	inf. temporal
fusiform	pars triangularis	inf. temporal	middle temporal
inf. temporal	rostr. mid. frontal	isthmus cingulate	post-central
lat. orbito-frontal	superior frontal	lat. occipital	pre-central
med. orbito-frontal	rh frontal pole	lingual	sup. temporal
middle temporal	rh insula	parahippocampal	supra-marginal
parahippocampal	**module 3 (green)**	post. cingulate	transv. temporal
pars orbitalis	cuneus	precuneus	insula
post. cingulate	lat. occipital	lh sup. parietal	rh inf. parietal
rostr. ant. cingulate	lingual	lh supra-marginal	rh pars opercularis
rostr. mid. frontal	pericalcarine	**module 5 (yellow)**	rh precuneus
sup. temporal	sup. parietal	inf. parietal	**module 7 (orange)**
temporal pole	rh precuneus	paracentral	caud. mid. frontal
insula		post-central	pre-central
lh pars opercularis		precuneus	superior frontal
lh pars triangularis		sup. parietal	rh paracentral
lh frontal pole		supra-marginal	

Supplementary Table S1: Module affiliation for the seven modules of the age-invariant structural correlation network. Names correspond to larger regions of the Desikan-Kiliany atlas (Desikan et al., 2006), of which our atlas is a sub-parcellation. A region name indicates that one of its sub-parcels belongs to the corresponding module; since each Desikan-Kiliany region contains multiple smaller parcels, the same region name can be listed under multiple modules. If no prefix is listed in front of a region name, the corresponding region is affiliated to the module on both hemispheres; else, lh = left hemisphere, rh = right hemisphere.

The von Economo atlas of cytoarchitectonic classes

The classification of regions into cytoarchitectonic classes was conducted manually based on the five original subtypes decribed by von Economo & Koskinas (1925); in order of increasing laminar differentiation, these correspond to primary motor cortex (structural type 1), association cortex (structural types 2 and 3) and secondary and primary sensory cortex (types 4 and 5). Our classification further distinguishes two additional subtypes - limbic regions and insular cortex, neither of which are easily assigned to the five original classes (Mai & Paxinos, 2012), resulting in seven cytoarchitectonic subtypes (Vértes et al., 2016). For a surface map with class labels, see **Fig. S2A**.

Functional intrinsic connectivity networks

We studied trajectories of structural network development relative to an independent cortical parcellation based on seven functional connectivity networks (Yeo, Krienen et al., 2011). We assigned the 308 regions in our parcellation to one of the seven classes based on the greatest proportion of overlap of each region to each class. The proportion of overlap was calculated in the *fsaverage* template volume per region as the number voxels within class, divided by the total number of voxels. For a surface map with class labels, see **Fig. S2B**.

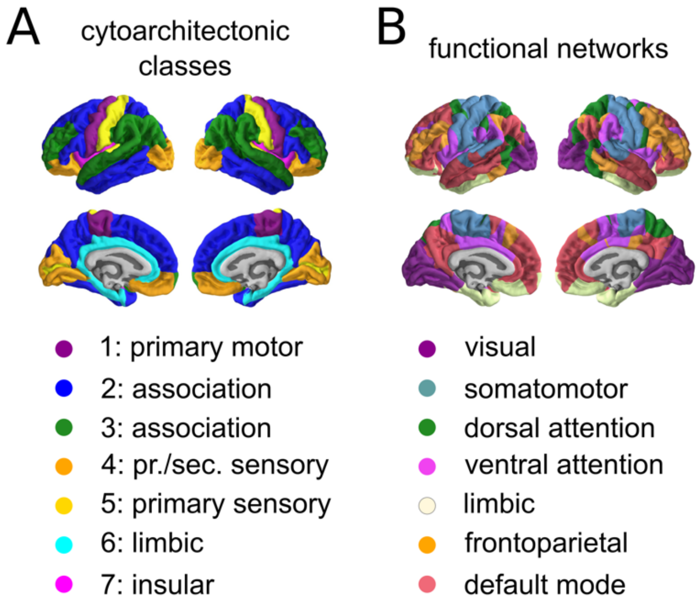

Supplementary Figure S2: Surface maps and community labels for two independent community templates. A) Cytoarchitectonic classes of the von Economo atlas (von Economo & Koskinas, 1925). B) Functional intrinsic connectivity networks (Yeo, Krienen et al., 2011).

Spatial permutation test

The spatial permutation test was implemented in the following manner: First, we obtained the coordinates of each of our 308 regions on the FreeSurfer spherical projection of the parcellation. We next rotated these coordinates about the three axes (x: left-right, y: rostral-caudal, z: dorsal-ventral) at three randomly generated angles, θ_x, θ_y and $\theta_z \in [0,2\pi)$, using the following rotation matrices:

$$R_x(\theta) = \begin{bmatrix} 1 & 0 & 0 \\ 0 & \cos(\theta) & -\sin(\theta) \\ 0 & \sin(\theta) & \cos(\theta) \end{bmatrix} \quad R_y(\theta) = \begin{bmatrix} \cos(\theta) & 0 & \sin(\theta) \\ 0 & 1 & 0 \\ -\sin(\theta) & 0 & \cos(\theta) \end{bmatrix} \quad R_z(\theta) = \begin{bmatrix} \cos(\theta) & -\sin(\theta) & 0 \\ \sin(\theta) & \cos(\theta) & 0 \\ 0 & 0 & 1 \end{bmatrix}$$

Since each hemisphere is projected onto the sphere separately, the rotation was applied to both hemispheres. To preserve hemispheric symmetry, the same random angles were applied to both hemispheres, with the caveat that the sign of the angles was flipped for the rotations around the y and z axes; i.e.:, $\theta_{yR} = -\theta_{yL}$ and $\theta_{zR} = -\theta_{zL}$ (but $\theta_{xL} = \theta_{xR}$).

Following rotation of the sphere, coordinates of the rotated regions were matched to coordinates of the initial regions, using Euclidean distance and proceeding in a descending order of average Euclidean distance between pairs of regions on the rotated and unrotated spheres (i.e.: starting with the rotated region that is furthest away, on average, from the unrotated regions). The matching then provides a mapping from the set of regions to itself, that allows any regional measure to be permuted while controlling for spatial contiguity and hemispheric symmetry.

P-values for the correlation between two maps were obtained by comparing the empirical value of Spearman's ρ to a null distribution of 10'000 Spearman correlations, between one empirical map and a set of 10'000 spatially permuted versions of the other map.

For an example outputs of the permutation test, see **Fig. S3** below.

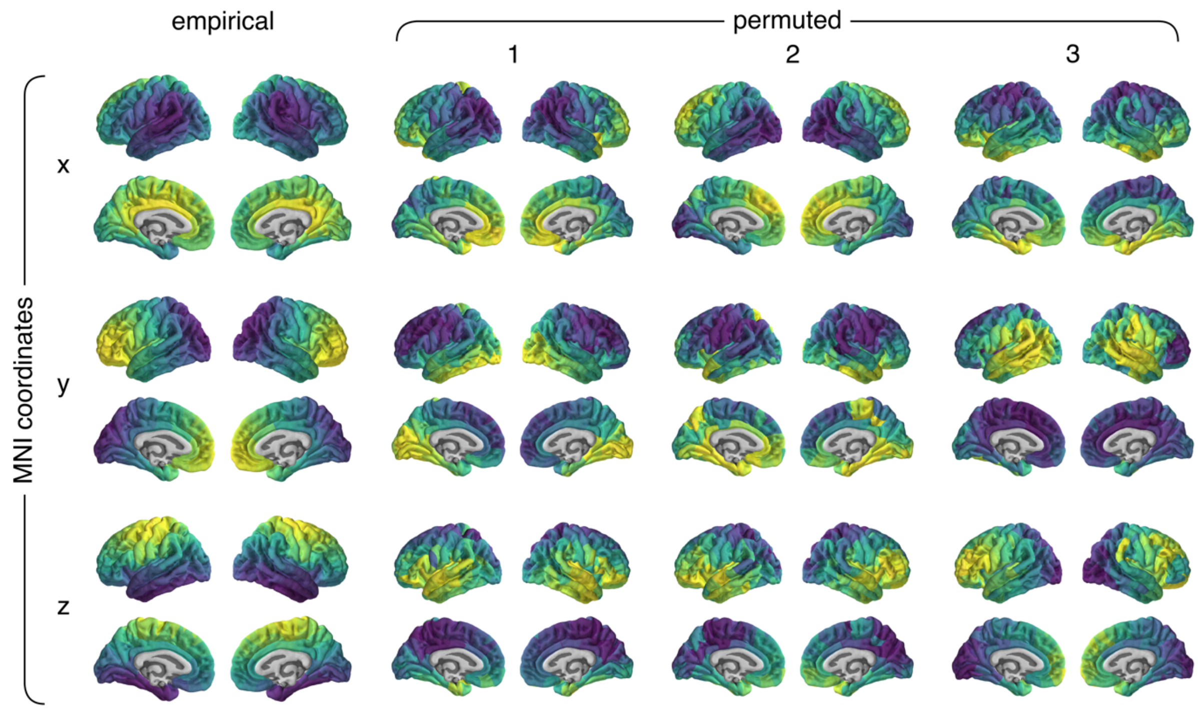

Supplementary Figure S3: Demonstration of the spatial permutation test. The spatial permutation test was applied three times to maps of the x, y and z coordinates. The spatial contiguity and hemispheric symmetry is preserved within the permuted maps.

Trajectories of structural correlation, covariance and variance

To ascertain more detail about trajectories of structural correlation, we investigated components of Pearson's correlation coefficient ($corr(x,y) = cov(x,y)/\sigma(x)\sigma(y)$), including the covariance ($cov(x,y)$, numerator of the correlation coefficient) and the (product of) variance(s) ($\sigma(x)\sigma(y)$, denominator of the correlation coefficient). We estimated both the covariance and variance across pairs of regional cortical thickness values for each sliding window, for both empirical data and across the 1000 bootstraps. For each measure, we visualised shifts in the distribution of values (**Fig. S4 A-C i**), as well as the mean (**Fig. S4 A-C ii**) and standard deviation (**Fig. S4 A-C iii**) of the measure across the upper triangular part of the corresponding matrices.

We found that trajectories of mean covariance and product of variances were qualitatively consistent with the trajectory mean correlation, exhibiting a nonlinear decrease followed by a slow linear increase (covariance: $AIC_{spl} < AIC_{lin}$, $r^2_{adj} = 0.61$, $p = 0.059$, $age_{min} = 19.9$ y; **Fig. S4Bii**; product of variances: $AIC_{spl} < AIC_{lin}$, $r^2_{adj} = 0.67$, $p = 0.035$, $age_{min} = 20.7$ y, **Fig. S4Cii**). The standard deviations (across edges) of the three sets of distributions did not show substantial evidence of change with age, but did show small qualitative differences – while the trajectory of the standard deviation of correlations appeared relatively flat ($AIC_{spl} < AIC_{lin}$, $r^2_{adj} = 0.55$, $p = 0.12$, **Fig. S4Aiii**), the standard deviations of both the covariance and the product of variances followed slow non-linear decreases (covariance: $AIC_{spl} < AIC_{lin}$, $r^2_{adj} = 0.42$, $p = 0.16$, $age_{min} = 20.0$ y; **Fig. S4Biii**; product of variances: $AIC_{spl} < AIC_{lin}$, $r^2_{adj} = 0.45$, $p = 0.18$, $age_{min} = 18.7$ y, **Fig. S4Ciii**).

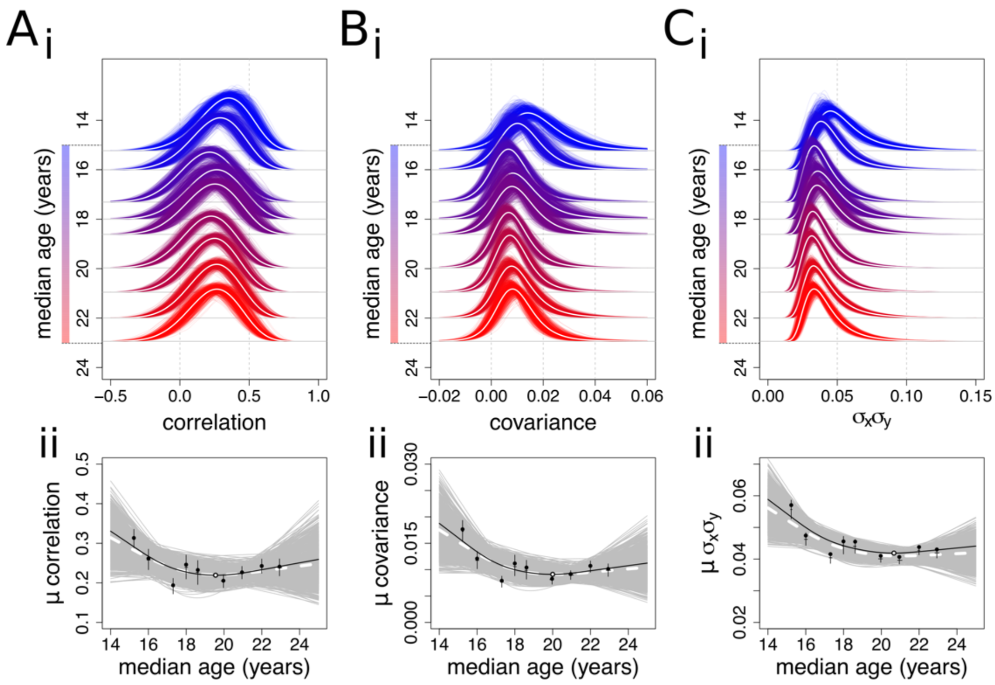

Supplementary Figure S4: Decomposition of structural correlation into structural covariance and the product of variances. Changes in the A) correlation, B) covariance and C) product of standard deviations of cortical thickness as both (i) distributions and (ii) means as a function of age. In panels (ii), black markers correspond to empirical data and spline fits, while grey lines correspond to trajectories of the bootstrapped correlations (with the white dashed line as their mean); vertical black lines correspond to inter-quartile ranges across bootstraps within each age-bin.

Unthresholded trajectories of node degree

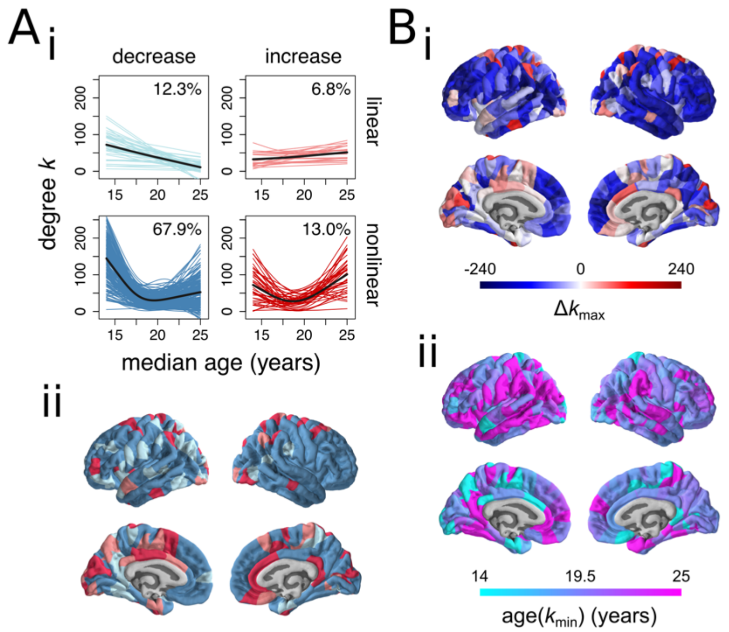

Supplementary Figure S5: Unthresholded regional trajectories of node degree within bootstrap-thresholded networks. A) (i) Classification of trajectories into linear or non-linear increase or decrease based on AIC (inset is in each plot is the percentage of regions in that category), and (ii) cortical location of each trajectory type (thin light lines correspond to trajectories of individual regions, thick dark lines to averages of each type). B) Regional properties of trajectories of node degree: (i) maximum change in degree $\boldsymbol{\Delta k_{max}}$, and (ii) age at minimum degree **age($\boldsymbol{k_{min}}$)**.

We classified regional changes in node degree as linear or non-linear (using the AIC), and increases or decreases (using the direction of maximum change; **Fig. S5A**). Most regions exhibited a nonlinear decrease (209/308 regions = 67.9%), followed by nonlinear increase (40/308 regions = 13.0%), linear decrease (38/308 regions = 12.3%) and finally linear increase (21/308 regions = 6.8%). The greatest decreases in node degree occurred in association cortex, which also reached minimum degree latest (**Fig. S5B**).

Unthresholded regional trajectories of correlation strength (in unthresholded networks)

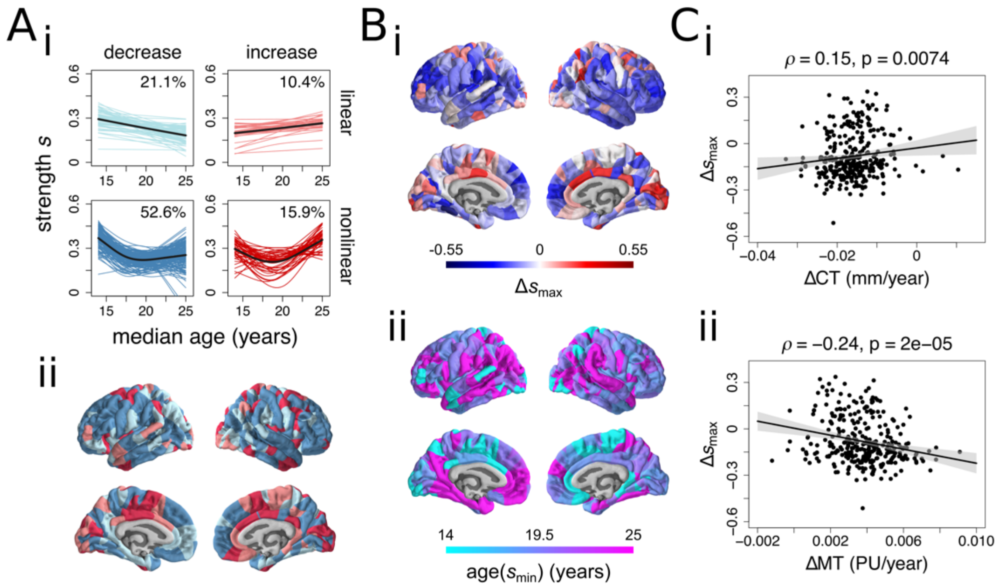

Supplementary Figure S6: Regional trajectories of nodal correlation strength within unthresholded networks. A) (i) Classification of trajectories into linear or non-linear increase or decrease based on AIC (inset in each plot is the percentage of regions in that category), and (ii) cortical location of each trajectory type (thin light lines correspond to trajectories of individual regions, thick dark lines to averages of each type). B) Regional properties of trajectories of node strength: (i) maximum change in strength Δs_{max}, and (ii) age at minimum strength **age(s_{min})**. C) Relationship of maximum change in strength to rates of cortical (i) thinning and (ii) myelination.

We classified regional changes in node strength as linear or non-linear (using the AIC), and increases or decreases (using the direction of maximum change; **Fig. S6A**). Most regions exhibited a nonlinear decrease (162/308 regions = 52.6%), followed by linear decrease (65/308 regions = 21.1%), nonlinear increase (49/308 regions = 15.9%) and finally linear increase (32/308 regions = 10.4%). As in the case of node degree, the greatest decreases in node strength occurred in association cortex, which also reached minimum degree latest (**Fig. S6B**). Further, as in the case of node degree, the maximum change in correlation strength was (weakly) positively correlated to the rate of thinning (ΔCT; $\rho = 0.15$, p = 0.0074; **Fig. S6C**), and (weakly) negatively correlated to the rate of myelination. In line with the magnitude of cortical myelination increasing as a function of increasing cortical depth until reaching a peak at 70% fractional depth, the (negative) relationship between developmental change in structural correlation and myelination was greatest at 70% cortical depth (ΔMT; $\rho = -0.24$, $p_{Spearman} = 2.0 \cdot 10^{-5}$, $p_{perm} = 0.013$; **Fig. S6B**). Overall, these relationships suggest that nodes that thin and myelinate faster from adolescence to early adulthood are more likely to decrease in structural correlation to other nodes, and conversely nodes that thin and myelinate slower over the same period are more likely to increase in structural correlation to other nodes. However, the small effect sizes suggest that adolescent trajectories of structural correlation networks contain information above trajectories of cortical morphology.

Trajectories of Euclidean distance

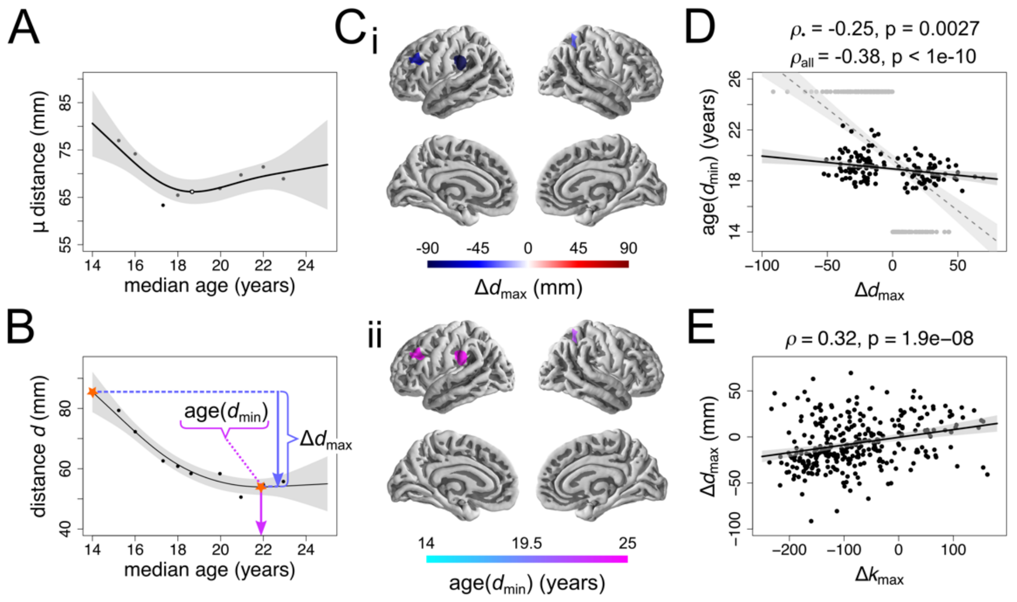

Supplementary Figure S7: Trajectories of Euclidean distance spanned by edges (retained after bootstrap-thresholding). A) Global trajectory of average Euclidean distance. B) Definition of local measures of maturation, illustrated on a nonlinearly decreasing trajectory (from the right superior parietal cortex). The maximum change in distance **Δ*d*$_{max}$** corresponds to the (absolute) difference (decrease or increase) in degree between the maximum and the minimum of the trajectory. The age at minimum distance **age(*d*$_{min}$)** corresponds to the timing of the minimum of the trajectory. C) Cortical maps of properties of regional trajectories of average Euclidean distance: (i) maximum change in distance **Δ*d*$_{max}$**, and (ii) age at minimum distance **age(*d*$_{min}$)**. D) Regions that show greater decreases in distance tend to reach minima of their trajectories later, whether considering all regions (grey) or excluding regions where the trajectory minimum occurs at extrema of the age range (black). E) Regions that show greater decreases in degree also show greater decreases in nodal connection distance.

The global connection distance of the thresholded networks (the mean Euclidean distance subtended by bootstrap-thresholded edges) demonstrated a non-linear trajectory (AIC_{lin} < AIC_{spl}, r^2_{adj} = 0.67, p = 0.049) characterised by a phase of relatively rapid decrease from 14 years to reach a minimum at about 18.7 years, followed by a phase of more stable connection distance (**Fig. S7A**). Nodes that demonstrated significantly reduced connection distance (p_{FDR} < 0.05) were located in left dorsolateral prefrontal cortex, left supramarginal gyrus and right superior parietal cortex (**Fig. S7C**). Decreases in node connection distance were negatively correlated with age at minimum connection distance, whether considering all nodes (Pearson's ρ = -0.38, $p_{Spearman} < 10^{-10}$, $p_{perm} = < 10^{-5}$) or excluding nodes whose minimum occurs at one of the limits of the age range (Pearson's ρ = -0.25, $p_{Spearman}$ = 0.0027, p_{perm} = 0.0036) (**Fig. S7D**).

Rates of thinning and myelination

Following Whitaker, Vértes et al. (2016), myelination (MT) analyses were conducted at 10 fractional depths between the pial surface and the grey/white matter boundary, as well as two absolute depths into white matter. The 10 intra-cortical samples of MT were chosen as fractions of cortical depth to adjust for differing cortical thicknesses between regions. For depths below the grey-white matter boundary, absolute depths were used to maintain uniformity across regions. The values of 0.4 mm and 0.8 mm were chosen for their correspondence to the spacing used for intra-cortical samples in the thickest cortical regions. For details on the extraction of MT values at variable cortical depths, see Whitaker, Vértes et al. (2016).

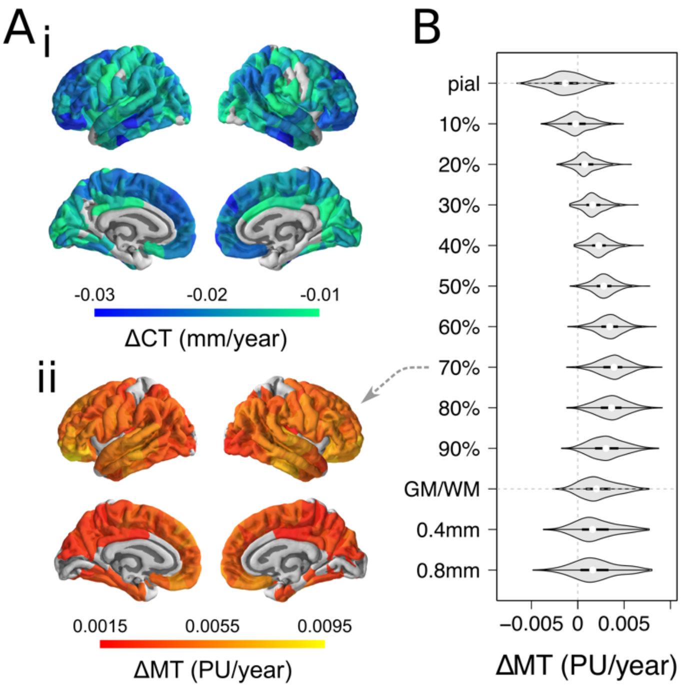

Supplementary Figure S8: Rates of thinning and myelination and derived results. A) Surface maps of rates of cortical (i) thinning and (ii) myelination (PU = percentage units). Only regions showing evidence of non-zero change (at $p_{FDR} < 0.05$) are shown. B) Rates of myelination across 308 regions as a function of cortical depth, including 10 fractional depths from the pial surface to the grey/white matter boundary (GM/WM), as well as two absolute depths into the white matter

As shown in Whitaker, Vértes et al. (2016), the greatest rates of thinning and myelination occur in association cortical areas (**Fig. S8A**), and the greatest rate of change of myelination occurs at 70% depth from the pial surface (**Fig. S8B**). We find that the relationship of (layer-specific) rate of myelination ΔMT and maximum change in degree is strongest at the same depth (**Fig. 4**).

Node degree within the age-invariant structural correlation network

The age-invariant network was thresholded using bootstrap, to retain all correlations consistent across resampling of all 297 participants with replacement (1000 samples, α_{FDR} = 0.01). Due to the high density of the thresholded age-invariant network (~90%), most nodes displayed very high degree (~75% of nodes have degree > 277, meaning they are at least 90% connected). Therefore, analyses involving node degree of the age-invariant structural correlation network were conducted using the weighted degree, or the node-wise sum of the weights of retained edges, which was better able to discriminate the importance of individual nodes.

Regions exhibiting highest (weighted) degree were located in frontal and occipital cortex (**Fig. S9A**). There is an inverse relationship between age-invariant weighted degree and maximum change in degree (ρ = -0.43, $p_{Spearman} < 10^{-10}$, $p_{perm} < 10^{-5}$; **Fig. S9Bi**), as well as weak evidence of a relationship between age-invariant weighted degree and age at minimum degree – whether all regions are considered (ρ = 0.10, $p_{Spearman}$ = 0.075, p_{perm} = 0.12) or only regions whose minimum of node degree does not occur at one of the extrema of the age range (ρ = 0.085, $p_{Spearman}$ = 0.23, p_{perm} = 0.15; **Fig. S9Bii**).

The relationships remain qualitatively consistent when binary node degree of the age-invariant network is used. There is an inverse relationship between age-invariant binary degree and maximum change in degree (ρ = -0.38, $p_{Spearman} < 10^{-10}$, $p_{perm} = < 10^{-5}$), as well as lack of evidence of a relationship between age-invariant binary degree and age at minimum degree – whether all regions are considered (ρ = 0.081, $p_{Spearman}$ = 0.16, p_{perm} = 0.080) or only regions whose minimum degree does not occur at one of the extrema of the age range (ρ = 0.023, $p_{Spearman}$ = 0.75, p_{perm} = 0.27).

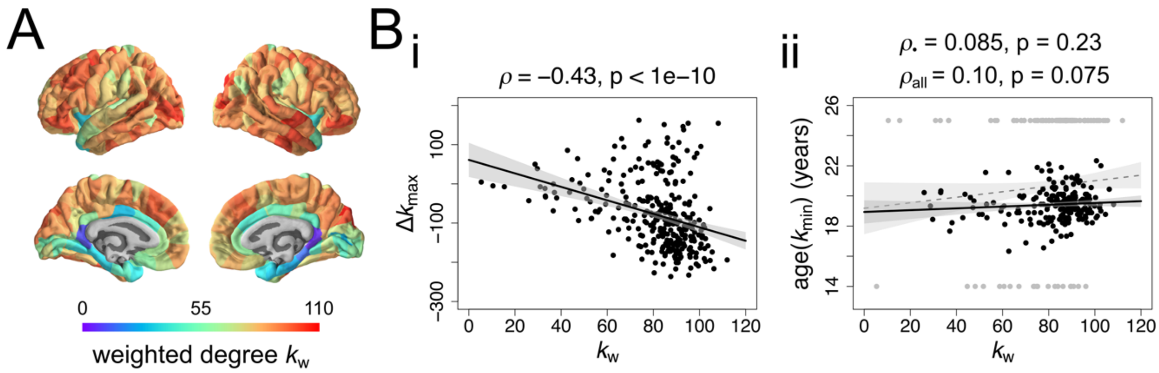

Supplementary Figure S9: Node degree within the age-invariant correlation network. A) Weighted node degree of the age-invariant correlation network, constructed using all 297 participants and thresholded using bootstrap. B) Correlation of weighted node degree within the age-invariant network and parameters of maturational change in adolescent correlation: (i) maximum change in node degree and (ii) age at minimum strength.

Linear rates of change of node degree

By fitting both linear and spline models to both trajectories, and comparing the quality of fit (given the parsimony of the model) using Akaike's Information Criterion (AIC), we have shown that the trajectories of node degree exhibited by most regions are best described as nonlinear (**Fig. 3** and **Fig. S5**). To estimate regional changes in degree, we have then used measures suitable for nonlinear models, including the maximum change in degree $\Delta \boldsymbol{k}_{\mathbf{max}}$ and the age at minimum degree **age(**$\boldsymbol{k}_{\mathbf{min}}$**)**. However, since (depending on the sliding window paramaters) the fitting of the nonlinear smoothing spline was conducted on relatively few data-points, we wished to ascertain that the results remain qualitatively consistent when the simplest possible model is used – the linear model, and the derived slope (the linear rate of change in degree $\Delta \boldsymbol{k}_{\mathbf{lin}}$).

Rates of linear change in node degree demonstrated greatest decreases in degree in association cortical areas (**Fig. S10A**), consistently with the nonlinear measure of maximum change in node degree. Further, linear rates of change of degree showed consistent direction and magnitude of correlation to rates of both cortical thinning (ΔCT; ρ = 0.20, $p_{Spearman}$ = 0.00048, p_{perm} = 0.037) and myelination (ΔMT; ρ = -0.33, $p_{Spearman}$ = $5.4 \cdot 10^{-9}$, p_{perm} = 0.0023).

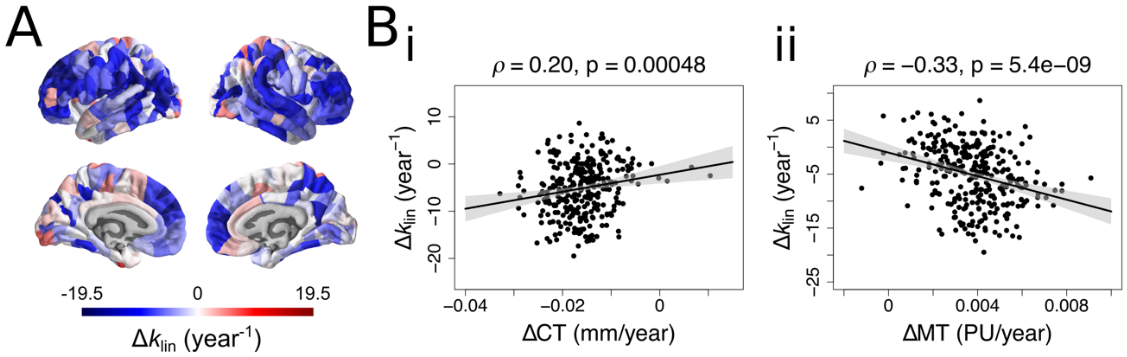

Supplementary Figure S10: Results are qualitatively consistent when fitting linear models to measure change in degree. A) Decreases in node degree, as measured using the linear rate of change of degree $\Delta \boldsymbol{k}_{\mathbf{lin}}$, remain greatest in association cortical areas. B) Correlations of rate of change of degree with rates of (i) thinning and (ii) myelination retain the same direction and magnitude as when using nonlinear measures of change in node degree.

Sensitivity Analyses

(In)dependence of results on sliding window parameters

The selection of sliding window parameters, including window width and step size (in units of number of participants) involves several trade-offs. On one hand, selecting a wider window increases the robustness of correlations within each of those windows, as they are estimated using more participants; on the other hand, the median ages of participants within each window will cover a narrower portion of the overall age-range. Furthermore, while a smaller step size will provide a greater density of windows and hence time-points for curve fitting and trajectory characterisation, a denser sampling of data will exacerbate issues with the inevitably uneven distribution of subjects across the age-range studied, which in effect corresponds to an unevenly sampled time-series. Specifically, while decreasing the step-size leads the method further from a discrete and closer to a continuous characterisation of the data, failure to take into account the uneven sampling introduces issues such as artefactual fluctuations in structural correlation related to local fluctuations in participant density. (For a discussion of this point, please see supplementary section "*Effects of irregularities in age-distribution of participants*" below.) Furthermore, with a denser sampling of participants, covariates might not be well conserved across windows, although this can potentially be controlled for using regression. Finally, with a smaller step size, the dependence between consecutive datapoints should technically be taken into account when quantitatively estimating effect sizes and p-values for the smooth trajectories of structural network properties.

Here we verify that our results are qualitatively robust to the choice of sliding window parameters, by repeating our core analyses across a range of values of window width ({40,60,80} participants) and step size ({5,10,20} participants). The resulting number of windows N_{wind} is calculated using the following equation:

$$N_{wind} = ceil((N_{part} - ww)/ss)$$

where N_{part} is the number of participants (here, 297), *ww* and *ss* are respectively the window width and step size (both in number of participants) and *ceil()* is the ceiling function, which rounds non-integer fractions up to the nearest integer (necessary for cases where due to the combination of parameters, one of the windows contains fewer participants than the rest). Trivially, the number of windows is inversely related to the window width and step size, such that greater windows and steps lead to fewer windows (and vice-versa). This affects the significance of model fits. However, the results confirm the robustness of our main findings (**Table S2** and **Fig. S11**).

Parameters			μ correlation r^2_{adj} (p)	edge density r^2_{adj} (p)	degree nonlinear $N(p_{FDR}<0.05)$	degree linear $N(p_{FDR}<0.05)$	ΔCT vs Δk_{max} ρ (p)	ΔMT vs Δk_{max} ρ (p)
**wind. width**	**step size**	**N. wind.**						
**60**	**30**	9	0.52 (0.098)	0.81 (0.0069)	75↓ 7↑	0↓ 0↑	0.16 (0.005)	-0.32 (6.6e-9)
**40**	**5**	52	0.40 (9.6e-5)	0.64 (<1e-10)	218↓ 55↑	5↓ 2↑	0.17 (0.0032)	-0.34 (<1e-10)
	**10**	26	0.39 (0.011)	0.64 (4.6e-5)	178↓ 44↑	5↓ 1↑	0.16 (0.0048)	-0.33 (5.4e-9)
	**20**	13	0.46 (0.055)	0.67 (0.0054)	105↓ 10↑	0↓ 0↑	0.081 (0.16)	-0.25 (7.4e-6)
**60**	**5**	48	0.47 (2.5e-5)	0.65 (4.7e-9)	185↓ 92↑	3↓ 2↑	0.19 (6.7e-4)	-0.32 (7.1e-9)
	**10**	24	0.48 (0.0052)	0.70 (1.5e-5)	175↓ 73↑	5↓ 5↑	0.20 (3.1e-4)	-0.33 (5.1e-9)
	**20**	12	0.58 (0.028)	0.75 (0.0028)	156↓ 26↑	0↓ 0↑	0.19 (8.2e-4)	-0.37 (<1e-10)
**80**	**5**	44	0.46 (8.5e-5)	0.48 (1.0e-4)	164↓ 113↑	2↓ 1↑	0.19 (6.2e-4)	-0.33 (4.1e-9)
	**10**	22	0.42 (0.017)	0.48 (0.011)	134↓ 96↑	4↓ 3↑	0.18 (0.0016)	-0.33 (2.5e-9)
	**20**	11	0.61 (0.030)	0.58 (0.047)	80↓ 39↑	0↓ 0↑	0.15 (0.0070)	-0.31 (2.2e-8)

Statistical significance		
p < 0.05	p < 0.01	p < 0.001

Supplementary Table S2: (In)dependence of results on sliding window parameters. Results from our main analyses, across main parameters (top row) and all combinations of window width and step sizes of {40,60,80} and {5,10,20} participants respectively. From left to right, the table lists the number of windows resulting from each combination of parameters, effect sizes and p-values for the mean (unthesholded) correlation and (thresholded) edge density, the number of regions showing evidence of nonlinear or linear increases or decreases in node degree (at $p_{FDR}<0.05$), as well as effect sizes and p-values of the relationship of maximum change in node degree to the rates of thinning and myelination. For columns where a statistical test was conducted, cell entries are colour-coded: p<0.001 in red, p<0.01 in orange and p<0.05 in yellow. Cells which are not considered statistically significant (p<0.05) or where no statistical test was carried out remain white.

The exact quantitative estimates of effect sizes and significance of fits vary across windows. Specifically, the greatest variation can be seen in the numbers of regions showing significant change with age (**Fig. S11 A-C iii,iv**) – the number of regions showing significant change increases with the number of windows. Furthermore, the edge density within each window varies across parameters, although the shape of the trajectory remains qualitatively consistent (**Fig. S11 A-C ii**). Finally, smaller steps between windows lead to local fluctuations in correlation, linked to fluctuations in the local edge density of participants (**Fig. S11 Ai**). However, this is somewhat mitigated by the probabilistic bootstrap-based thresholding, which eliminates inconsistent edges (**Fig. S11 Aii**). Our choice of parameters for the main analysis was designed to minimise overlap of neighbouring windows and maximise the conservation of co-variates across windows.

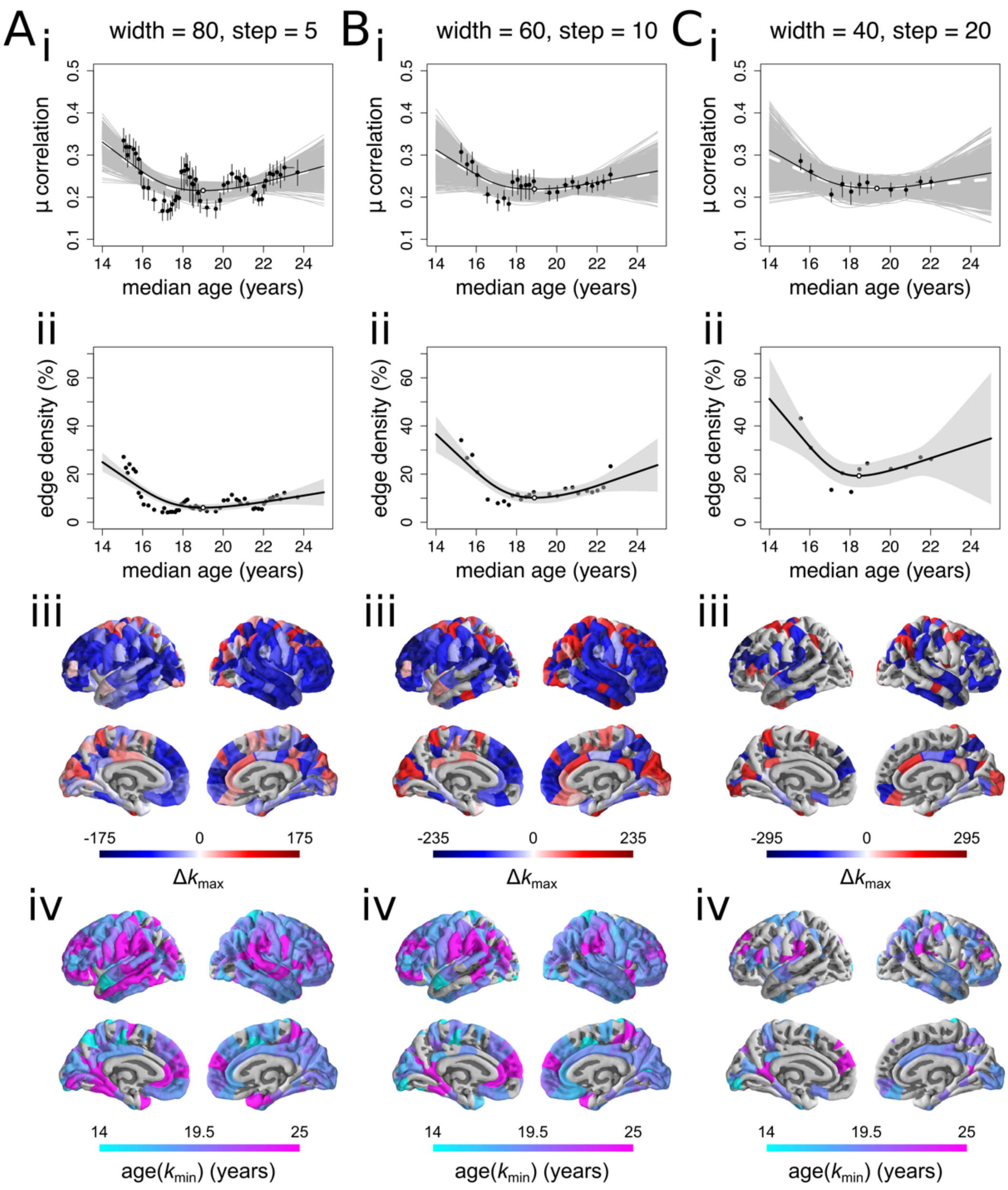

Supplementary Figure S11: (In)dependence of results on sliding window parameters. Selection of parameters, including A) window width of 80 participants with a step size of 5 participants, B) window width of 60 participants with a step size of 10 participants and C) window width of 40 participants with a step size of 20 participants. Results shown are: (i) Development of mean correlation (in unthresholded networks). (ii) Development of edge density (in bootstrap thresholded networks). (iii-iv) Local measures of (iii) maximum change in degree $\boldsymbol{\Delta k_{max}}$ and (iv) age at minimum degree $\textbf{age}(\boldsymbol{k_{min}})$, for regions whose trajectories satisfy $p_{FDR} < 0.05$.

Effects of gender

Analysis of gender differences failed to show effects of gender or an age-by-gender interaction at the global level, within the mean correlation (p_{gender} = 0.49, p_{inter} = 0.97) or within the edge density of the thresholded networks (p_{gender} = 0.54, p_{inter} = 0.94). At the nodal level, after FDR adjustment for multiple comparisons (α_{FDR} = 0.05), four regions showed an effect of gender, and 16 regions showed evidence of an age-by-gender interaction. However, due to the lack of spatial clustering of these results, and inconsistency of the gender-specific trajectories across regions, these results are not compelling.

Effects of regional reliability

To ascertain the robustness of the obtained trajectories and to rule out potential artefactual causes, we conducted control experiments of the obtained trajectories. Many of these consisted in identifying sets of regions which could have been implicated in a particular artefact, and comparing trajectories averaged within and outside of these “masks”. We expected to see similar qualitatively similar changes with age in both resulting trajectories as evidence of the lack of influence of the potentially artefactual regions on the results.

First, we wished to verify whether estimation of structural correlations was more reliable in certain regions than others, and whether this affected results. Regional reliability was evaluated as the average similarity between regional correlation patterns generated from repeated random half-splits of the data. Specifically, we randomly subdivided subjects into two groups of equal size, built two structural correlation matrices by cross-correlating cortical thickness within each random half-group, and defined regional reliability as the correlation between the patterns of correlations (weights) of a region across the two splits (i.e.: correlations between rows – or equally, columns – of the resulting two matrices). Maps of reliability averaged over 1000 splits were thresholded using cross-correlations between random permutations of regions (the average reliability expected by chance), giving a set of “reliable” and “unreliable” regions. These two sets were used as masks, within which both empirical and bootstrapped correlations were averaged, to evaluate the effects of regional reliability on the developmental trajectories of structural correlations.

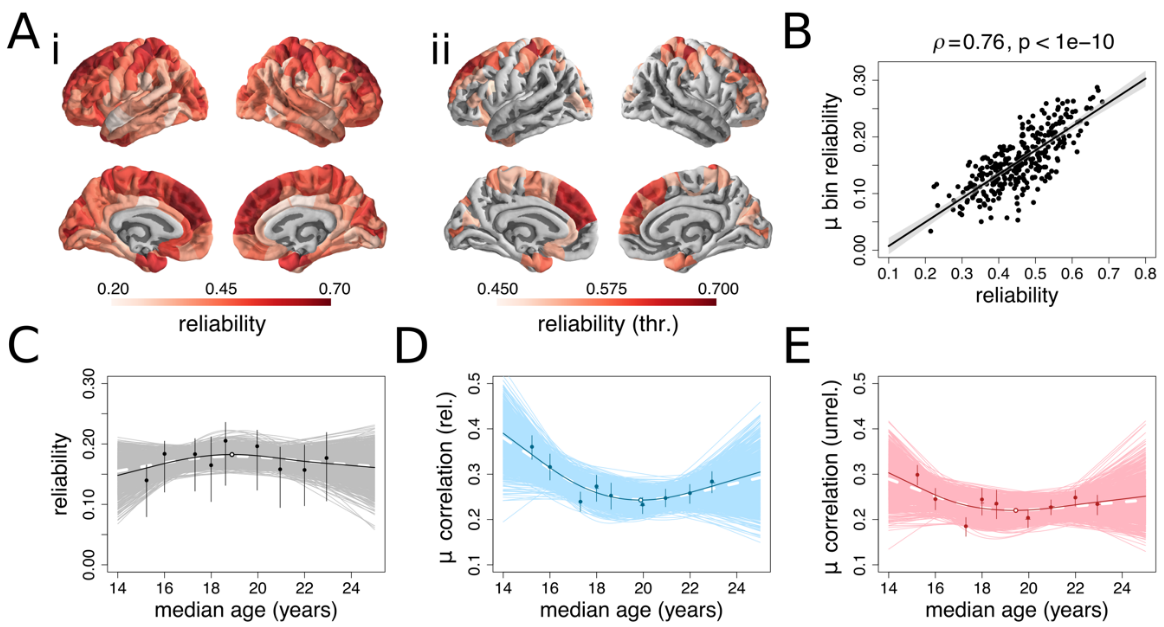

Supplementary Figure S12: Effects of regional reliability on trajectories of structural correlations. A) (i) Reliability of regional cortical thickness correlations, estimated as the average correlation between rows of pairs of correlation matrices generated from 1000 random half-splits of the data. (ii) "Reliable" regions, thresholded using random permutation of regions (rows) across the random half-splits (104/308 regions = 33.8%). B) When repeated within each (sliding) window, the average reliability is highly correlated to the overall (static) reliability. This, together with weak evidence of change in regional reliability across windows (C), justifies using reliable regions (Aii) as a mask to investigate the effect of regional reliability on structural correlations. D) The mean correlation within reliable regions is higher, shows a greater magnitude of change and a more nonlinear trajectory than E) the mean correlation within the remaining regions.

Results show a pattern of reliable regions located predominantly in superior frontal cortex and postcentral gyrus (**Fig. S12A**), in similar locations to regions reported as most reliable during estimation of cortical thickness (Iscan et al., 2015; Liem et al., 2015; Madan & Kensinger, 2017) (with the exception of high reliability of structural correlations in inferior temporal cortex in our data, likely due to the manual correction of our data in these known "dropout" locations). When estimated within sliding-window bins, the reliability is, on average, highly correlated to the overall reliability (**Fig. S12B**), which together with weak evidence of changes in structural correlation reliability (r^2_{adj} = 0.12, p = 0.51; **Fig. S12C**) justifies using the "overall reliable" regions as a mask. The trajectory of average structural correlations within these "reliable" regions shows both greater evidence of change as well as a more nonlinear trajectory (r^2_{adj} = 0.81, p = 0.0073, *df* = 3.3; **Fig. S12D**) than the trajectory of change within the remaining "unreliable" regions (r^2_{spl} = 0.31, p = 0.27, *df* = 3.1; **Fig. S12E**) (Wilcoxon Rank-sum test of difference in *df* across bootstraps: $p < 10^{-10}$). We conclude that the nonlinear decrease in structural correlation isn't driven by a potential unreliability of structural correlation estimates; if anything, it might be attenuated by certain less reliable regions. The reliability of structural correlation estimates remains a topic for further study.

Effects of irregularities in age-distribution of participants

Although the NSPN study was carefully designed to sample a uniform distribution of participants over age, there remain unavoidable small irregularities in age difference between consecutive participants (seen in the plot of participant age as a function of participant rank, **Fig. S13A**; a perfectly uniform distribution of participants across age would lead to a straight line). To evaluate the potential effect of these irregularities on results, we calculated age differences between consecutive participants and averaged them within age-bins. We then cross-correlated the resulting time-course (**Fig. S13B**) with regional time-courses of node strength (as a function of age), leading to an estimate of the proportion of variance in trajectory fluctuation (with age) explained by local non-uniformities in the age distribution of participants (**Fig. S13C**). Further, we divided regions into two groups – those "affected" regions for which age-difference-fluctuations explained more than 10% variance (over time), and the remaining "unaffected" regions. We used these regions as masks, within which both empirical and bootstrapped correlations were averaged.

Regions affected by the fluctuations are not spatially clustered, nor are they prominently located in association cortex (where the greatest decreases in correlation strength and node degree were found) (**Fig. S13C**). Following averaging of structural correlation within subsets of regions, regions that are only weakly affected by fluctuations in the distribution of participants show greater evidence for change as well as more nonlinear trajectories (r^2_{adj} = 0.61, p = 0.064, *df* = 3.26; **Fig. S13D**) than regions which are more strongly affected by these fluctuations (r^2_{adj} = 0.33, p = 0.22, *df* = 2.95; **Fig. S13E**) (Wilcoxon Rank-sum test of difference in *df* across bootstraps: $p < 10^{-10}$). Therefore, we conclude that the nonlinear decrease in structural correlation isn't driven by potential artefacts related to small irregularities in the age-distribution of participants; if anything, it might be attenuated by this.

It should be noted that these artefacts cannot appropriately be controlled for using interpolation, which is the simplest method for dealing with unevenly distributed data. However, future development of tools for the analysis of unevenly sampled time-series (Eckner, 2014) will hopefully alleviate such issues.

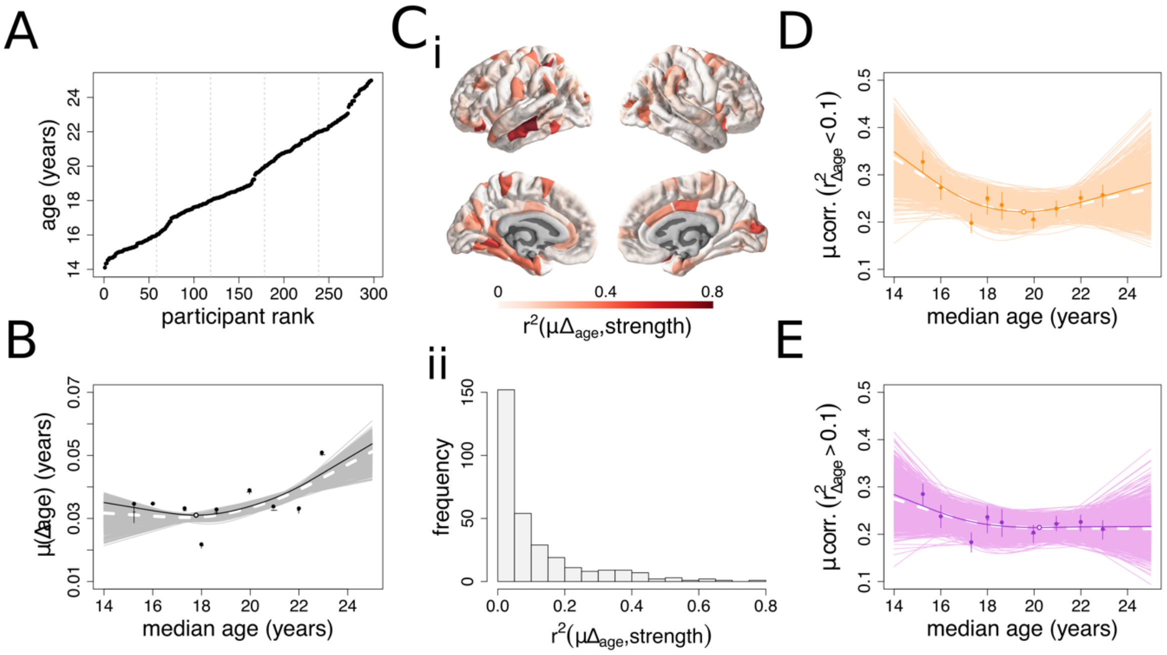

Supplementary Figure S13: Effects of inhomogeneity in the age-distribution of participants. A) While participants are sampled very uniformly, there remain inevitable small inhomogeneities in the age-gaps between consecutive participants (a perfectly homogenous distribution of participants with age would result in a straight line). B) There is a difference in average gaps between consecutive participants across the age-range, such that later windows show a greater gap between consecutive participants. C) Cross-correlating the pattern of age-gaps between consecutive participants with nodal strength yields several regions within which a high proportion of variance in strength with age appears to be caused by the age-inhomogeneities. (ii) However, most regions are only weakly correlated to this pattern. D) Averaging structural correlations within regions whose pattern of node strength fluctuations is relatively unaffected by age inhomogeneities (arbitrary cut-off at $r^2(str,\Delta_{age}) < 0.1$; 206/308 regions = 66.9%) leads to more non-linear trajectories that present greater magnitudes of change than E) averaging structural correlations within regions whose pattern of node strength fluctuations is more strongly affected by age inhomogeneities (arbitrary cut-off at $r^2(str,\Delta_{age}) > 0.1$; 102/308 regions = 33.1%).

Effects of non-linearities in trajectories of cortical thickness and myelination

Although trajectories of cortical thinning (and myelination) have been reported as linear in the current (cross-sectional) dataset (Whitaker, Vértes et al., 2016), subtle non-linearities may be present in the data, driving the nonlinear trajectories of structural correlation (and derived measures of edge density and node degree). To rule out effects of subtle non-linearities in trajectories of cortical thinning (and myelination) on trajectories of structural correlation, we fitted (potentially non-linear) smoothing splines to trajectories of both cortical thickness and myelination at all 308 regions, using data from all 297 participants included in this study. We compared the quality of linear and smoothing spline fits, given the parsimony of these models, using Akaike's Information Criterion (AIC), and extracted the degrees of freedom (*df*) of the smoothing spline models, indicative of the amount of non-linearity in the fit. A trajectory with *df* = 2 is linear (the two degrees of freedom in this case correspond to its intercept and slope), and as the number of degrees of freedom increases, the trajectory becomes more non-linear. We note that we used the same smoothing spline models as for structural network trajectories (see methods), constraining the trajectories to be approximately as smooth as quadratic models (i.e.: effective degrees of freedom $df \leq 3.5$).

Nonlinearities in cortical thickness

Within cortical thickness data, values of AIC were in general very similar for the linear model and smoothing splines. Although 203/308 regions (65.9%) displayed a better fit with the smoothing spline ($AIC_{spl} < AIC_{lin}$), most of these trajectories remained quasi-linear – the distribution of degrees of freedom remained heavily skewed towards 2 (**Fig. S14A**), with only 112/308 regions (36.4%) displaying weak evidence of nonlinearity ($df_{CT} > 2.01$; **Fig. S14B**). Inspecting examples of the 10 "most linear" and 10 "most non-linear" trajectories (**Fig. S14C,D**) indicates heterogeneity in the non-linearities, with few trajectories showing a similar shape of trajectory to structural correlation. Subsequently, averaging structural correlation within the 196/308 regions (63.6%) showing linear thinning ($df_{CT} = 2$; grey in **Fig. S14B**) led to similarly nonlinear trajectories ($r^2_{adj} = 0.53$, p = 0.099, *df* = 2.76; **Fig. S14E**) as averaging structural correlation within the remaining 112/308 (36.4%) "non-linear" regions ($r^2_{adj} = 0.55$, p = 0.083, *df* = 2.76; **Fig. S14F**). In fact, across bootstraps, trajectories of structural correlation within regions showing subtle non-linearities in cortical thinning had *lower* median *df* (2.82) than regions showing purely linear trajectories of thinning (median *df* = 3.03; Wilcoxon rank-sum test $p = 1.29 \cdot 10^{-5}$). This indicates that subtle non-linearities in trajectories of cortical thinning are not driving our main finding, of a nonlinear trajectory of structural correlation.

Additionally, we have verified that the degrees of freedom of cortical thickness trajectories df_{CT} do not correlate to the degrees of freedom of trajectories of nodal correlation (Spearman $\rho = 0.024$, $p_{Spearman} = 0.68$, $p_{perm} = 0.26$), the degrees of freedom of trajectories of node degree (Spearman $\rho = 0.054$, $p_{Spearman} = 0.34$, $p_{perm} = 0.11$), the maximum change in degree Δk_{max} (Spearman $\rho = -0.072$, $p_{Spearman} = 0.20$, $p_{perm} = 0.11$) or the age at minimum degree age(k_{min}) (Spearman $\rho = 0.0012$, $p_{Spearman} = 0.98$, $p_{perm} = 0.57$). This further indicates that subtle non-linearities in trajectories of cortical thinning are not influencing our regional results.

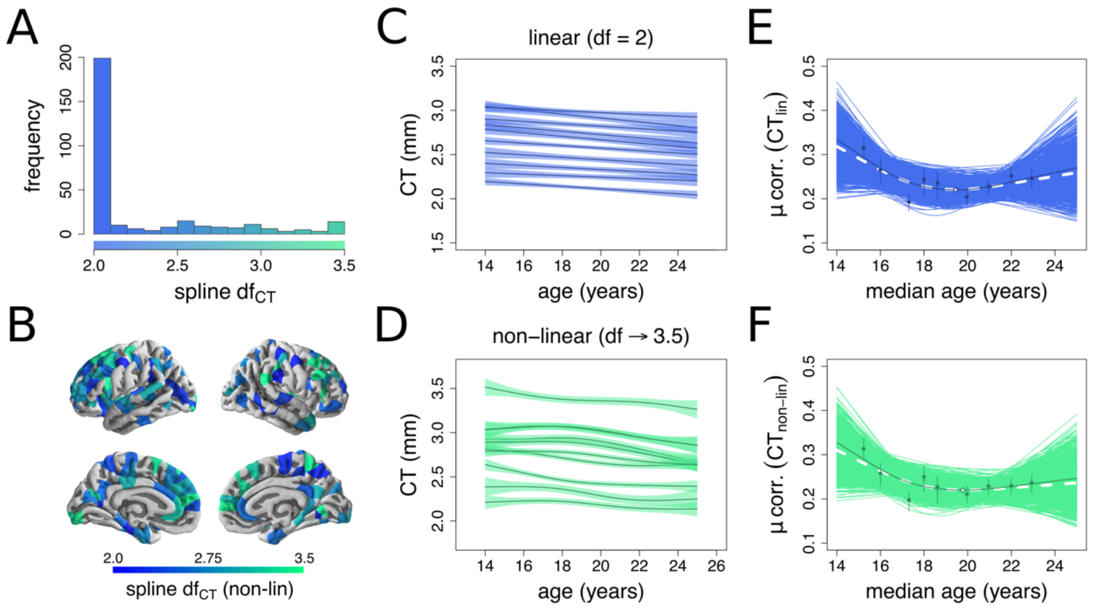

Supplementary Figure S14: Effects of subtle non-linearities in cortical thickness trajectories. A) The distribution of nodal degrees of freedom for spline trajectories of cortical thinning. Most regions show linear trajectories, with df_{CT} = 2. B) Cortical surface plots for nodal degrees of freedom, for the subset of regions showing subtle non-linearities (df_{CT} > 2.01); regions showing linear thinning (df_{CT} = 2) are plotted in gray. E) Averaging structural correlations within regions showing purely linear trajectories (df_{CT} = 2; 196/308 regions = 63.6%) leads to equally non-linear trajectories (of structural correlation) as averaging structural correlations within regions showing subtle non-linearities (df_{CT} > 2.01; 112/308 regions = 36.4%).

Nonlinearities in myelination

Further, we repeated the above analyses, inspecting subtle non-linearities in trajectories of cortical myelination. As in the main analyses and as in (Whitaker, Vértes et al., 2016), we focused on myelination estimated at approximately 70% depth between the pial surface and the grey / white matter boundary.

For trajectories of myelination, values of AIC were again very similar for the linear model and smoothing splines. Although 236/308 regions (76.6%) displayed a better fit with the smoothing spline (AIC_{spl} < AIC_{lin}), most of these trajectories remained quasi-linear – the distribution of degrees of freedom remained heavily skewed towards 2 (**Fig. S15A**), with only 115/308 regions (37.3%) displaying weak evidence of nonlinearity (df_{MT} > 2.01; **Fig. S15B**). Inspecting examples of the 10 "most linear" and 10 "most non-linear" trajectories (**Fig. S15C,D**) indicates greater homogeneity in the non-linearities than trajectories of cortical thickness; still, few trajectories show a similar shape of trajectory to our main trajectory of structural correlation. Subsequently, averaging structural correlation within the 193/308 regions showing linear myelination (df_{MT} = 2; grey in **Fig. S15B**) led to similarly nonlinear trajectories (r^2_{adj} = 0.58, p = 0.075, *df* = 2.76; **Fig. S15E**) as averaging structural correlation within the remaining 115/308 (37.3.6%) "non-linear" regions (r^2_{adj} = 0.51, p = 0.083, *df* = 2.76; **Fig. S15F**). Interestingly, across bootstraps, trajectories of structural correlation within

regions showing subtle non-linearities in cortical myelination had *higher* median *df* (3.00) than regions showing purely linear trajectories of myelination (median *df* = 2.88; Wilcoxon rank-sum test p = 0.036). This indicates that subtle non-linearities in trajectories of cortical myelination may be (weakly) related to our main finding, of a nonlinear trajectory of structural correlation. The idea that myelination is a stronger driver of structural correlation than cortical thinning would also be supported by our finding that the maximum change in degree Δk_{max} is more strongly correlated to the rate of cortical myelination (ΔMT) than thinning (ΔCT).

Additionally, we have verified whether degrees of freedom of myelination trajectories df_{MT} correlate with measures derived from (nonlinear) trajectories of structural correlation. The correlations were weak for the degrees of freedom of trajectories of nodal correlation (Spearman ρ = -0.013, $p_{Spearman}$ = 0.82, p_{perm} = 0.37), the degrees of freedom of trajectories of node degree (Spearman ρ = 0.024, $p_{Spearman}$ = 0.68, p_{perm} = 0.47) and the maximum change in degree Δk_{max} (Spearman ρ = 0.015, $p_{Spearman}$ = 0.79, p_{perm} = 0.40) but stronger for the age at minimum degree age(k_{min}) (Spearman ρ = -0.14, $p_{Spearman}$ = 0.016, p_{perm} = 0.059). This further indicates that subtle non-linearities in trajectories of cortical myelination may have a stronger influence on structural correlation than subtle non-linearities in trajectories of cortical thinning.

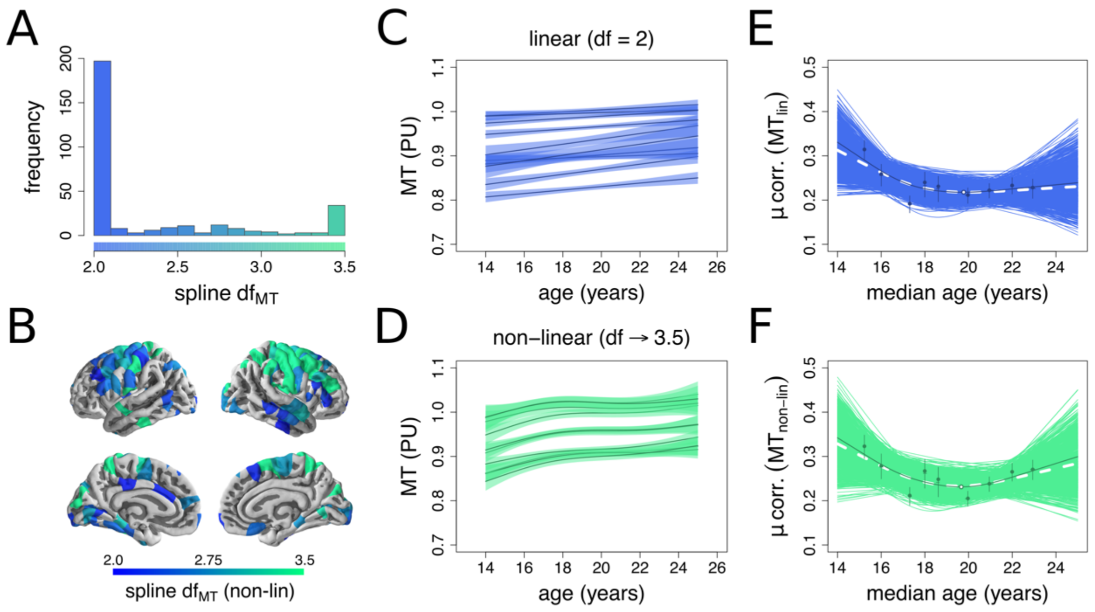

Supplementary Figure S15: Effects of subtle non-linearities in myelination trajectories. A) The distribution of nodal degrees of freedom for spline trajectories of cortical myelination. Most regions show linear trajectories, with df_{MT} = 2. B) Cortical surface plots for nodal degrees of freedom, for the subset of regions showing subtle non-linearities (df_{MT} > 2.01); regions showing linear thinning (df_{MT} = 2) are plotted in gray. E) Averaging structural correlations within regions showing purely linear trajectories (df_{MT} = 2; 196/308 regions = 63.6%) leads to equally non-linear trajectories (of structural correlation) as averaging structural correlations within regions showing subtle non-linearities (df_{MT} > 2.01; 112/308 regions = 36.4%).

Sub-sample analysis

To further investigate potential effects of non-linearities in trajectories of cortical thinning on nonlinear trajectories of structural correlation, we fitted linear models separately to the youngest and oldest halves of our participants (respectively 149 participants (75 female), aged 14.10-18.63 years, and 148 participants (74 female) aged 18.64-24.98 years).

We find that the rate of cortical thinning is generally slower in the first (younger) half of the sample (**Fig. S16A**) than in the second (older) half (**Fig. S16B**), with the “acceleration of thinning” occurring fastest in association cortex (**Fig. S16C**). These results agree with a previous longitudinal study, showing an acceleration of cortical thinning in adolescence (Zhou et al., 2015). Conversely, rates of myelination are faster in the first (younger) half-sample (**Fig. S16D**) than in the second (older) half (**Fig. S16E**), although in this case the difference is greatest in superior parietal regions (**Fig. S16F**).

The location of the greatest differences in rates of thinning (**Fig. S16C**) and myelination (**Fig. S16F**) between the two half-samples align with the locations of greatest non-linearities in these trajectories (respectively **Fig. S14B** and **Fig. S15B**); while the trajectory of cortical thinning demonstrates a faster rate of change in the second half of the age-range, the trajectory of myelination shows a faster rate of change in the first half of the age range. The differences in slope in **Fig. S16 C** and **F** are both negative due to the signs of the trajectories; in the case of thinning (where both slopes are negative) this corresponds to a *faster decrease* in thickness in the second half of the age range (**Fig. S16C**), whereas in the case of myelination (where both slopes are positive) this corresponds to a *slower increase* in myelination in the second half of the age range than in the first (**Fig. S16F**).

Thus, the differences in the rates of thinning between the two half samples differ from the trajectory of structural correlation, which shows a faster rate of change (decrease) in the first half of our age-range, than in the second half (where it levels off or even slightly increases). The fact that myelination is faster in the first half than in the second shows closer alignment with trajectories of structural correlation; however, the loci of these effects on the cortex differ. Thus, this analysis provides further evidence that the non-linearity within trajectories of structural correlation is not entirely driven by subtle non-linearities within trajectories of cortical thinning and myelination – although it appears more strongly related to the latter than to the former.

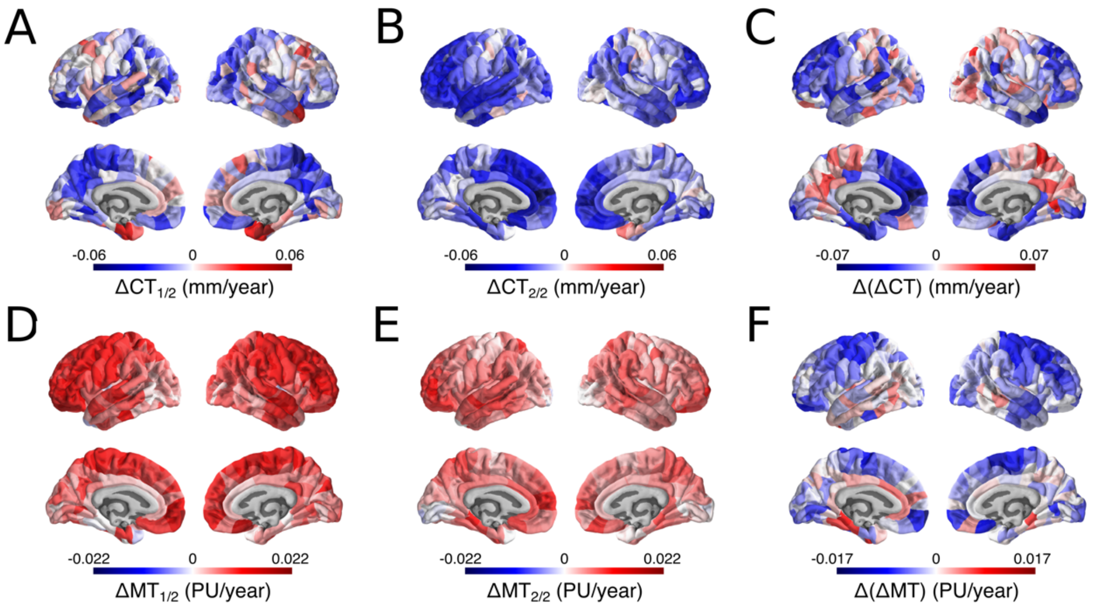

Supplementary Figure S16: Rates of thinning and myelination in sub-samples of participants. Top row: The rate of thinning in the first (A) and second (B) half-samples of participants, and the difference between the two (C). Bottom row: The rate of myelination in the first (D) and second (E) half-samples of participants, and the difference between the two (F).

Neuroscience in Psychiatry Network (NSPN) Consortium author list

Principal investigators:
Edward Bullmore (chief investigator from 01/01/2017)
Raymond Dolan
Ian Goodyer (chief investigator until 01/01/2017)
Peter Fonagy
Peter Jones

NSPN (funded) staff:
Michael Moutoussis
Tobias Hauser
Petra Vértes
Kirstie Whitaker
Gita Prabhu
Laura Villis
Junaid Bhatti
Becky Inkster
Cinly Ooi
Barry Widmer
Ayesha Alrumaithi
Sarah Birt
Kalia Cleridou
Hina Dadabhoy
Sian Granville
Elizabeth Harding
Alexandra Hopkins
Daniel Isaacs
Janchai King
Danae Kokorikou
Harriet Mills
Ciara O'Donnell
Sara Pantaleone

Affiliated scientists:
Pasco Fearon
Anne-Laura van Harmelen
Rogier Kievit

Supplementary References

Desikan, R. S., Ségonne, F., Fischl, B., Quinn, B. T., Dickerson, B. C., Blacker, D., … Killiany, R. J. (2006). An automated labeling system for subdividing the human cerebral cortex on MRI scans into gyral based regions of interest. *Neuroimage*, *31*, 968–980.

Eckner, A. (2014). Some Properties of Operators for Unevenly Spaced Time Series. Retrieved from http://www.eckner.com/research.html

Iscan, Z., Jin, T. B., Kendrick, A., Szeglin, B., Lu, H., Trivedi, M., … Delorenzo, C. (2015). Test-retest reliability of freesurfer measurements within and between sites: Effects of visual approval process. *Hum. Brain Mapp.*, *36*, 3472–3485.

Lancichinetti, A., & Fortunato, S. (2012). Consensus clustering in complex networks. *Sci. Rep.*, *2*. doi:10.1038/srep00336

Liem, F., Mérillat, S., Bezzola, L., Hirsiger, S., Philipp, M., Madhyastha, T., & Jäncke, L. (2015). Reliability and statistical power analysis of cortical and subcortical FreeSurfer metrics in a large sample of healthy elderly. *Neuroimage*, *108*, 95–109.

Madan, C. R., & Kensinger, E. A. (2017). Test–retest reliability of brain morphology estimates. *Brain Informatics*, 1–15.

Mai, J. K., & Paxinos, G. (2012). *The human nervous system*. Elsevier Academic Press.

Shinn, M., Romero-Garcia, R., Seidlitz, J., Váša, F., Vértes, P. E., & Bullmore, E. (2017). Versatility of nodal affiliation to communities. *Sci. Rep.*, *7*. doi:10.1038/s41598-017-03394-5

Vértes, P. E., Rittman, T., Whitaker, K. J., Romero-Garcia, R., Váša, F., Kitzbichler, M. G., … Ji, S. (2016). Gene transcription profiles associated with inter-modular hubs and connection distance in human functional magnetic resonance imaging networks. *Philos. Trans. R. Soc. Lond. B. Biol. Sci.*, *371*, 735–769.

von Economo, C., & Koskinas, G. N. (1925). *Die Cytoarchitektonik der Hirnrinde des Erwachsenen Menschen: Textband und Atlas mit 112 Mikrophotographischen Tafeln*. Springer, Vienna.

Weiskopf, N., Suckling, J., Williams, G., Correia, M. M., Inkster, B., Tait, R., … Lutti, A. (2013). Quantitative multi-parameter mapping of R1, PD(*), MT, and R2(*) at 3T: a multi-center validation. *Front. Neurosci.*, *7*, 95.

Whitaker, K. J., Vértes, P. E., Romero-Garcia, R., Váša, F., Moutoussis, M., Prabhu, G., … Bullmore, E. T. (2016). Adolescence is associated with transcriptionally patterned consolidation of the hubs of the human brain connectome. *Proc. Natl. Acad. Sci. U. S. A.*, *113*, 9105–9110.

Yeo, B. T. T., Krienen, F. M., Sepulcre, J., Sabuncu, M. R., Lashkari, D., Hollinshead, M., … Buckner, R. L. (2011). The organization of the human cerebral cortex estimated by intrinsic functional connectivity. *J. Neurophysiol.*, *106*. doi:10.1152/jn.00338.2011

Zhou, D., Lebel, C., Treit, S., Evans, A., & Beaulieu, C. (2015). Accelerated longitudinal cortical thinning in adolescence. *Neuroimage*, *104*, 138–145.
